# Electroadhesion in Polymer–Polymer Systems: From Theory and Modeling to Emerging Applications

**DOI:** 10.3390/polym18182263

**Published:** 2026-09-16

**Authors:** Konstantin I. Sharov, Tatiana N. Popyrina, Uliana V. Nikulova, Aleksey V. Shapagin

**Affiliations:** 1 Frumkin Institute of Physical Chemistry and Electrochemistry, Russian Academy of Sciences (IPCE RAS), 31-4, Leninsky Prospect, Moscow 119071, Russia; deathknight_91@mail.ru (K.I.S.); tanjapopyrina@yandex.ru (T.N.P.); ulianan@rambler.ru (U.V.N.); 2 Kurchatov Complex of Crystallography and Photonics, NRC “Kurchatov Institute”, 1 Academician Kurchatov Square, Moscow 123182, Russia; 3 Moscow Aviation Institute, National Research University, 4 Volokolamskoe sh., Moscow 125993, Russia

**Keywords:** electroadhesion, polymer–polymer systems, electroadhesion theory, electrode geometry, parameters influencing electroadhesion, electroadhesion application

## Abstract

This review provides a comprehensive analysis of the current state of electroadhesion research and the development of electroadhesive systems. Over one hundred scientific references were analyzed, covering the theoretical frameworks and modeling of electroadhesion, electroadhesive manufacturing technologies, factors influencing electroadhesive interactions, and the development prospects and applications of electroadhesion. The necessity for a detailed molecular-level investigation into polymer structures is substantiated to improve the accuracy of engineering forecasting and the integration of this technology in robotics, medicine, and autonomous systems based on triboelectric nanogenerators.

## 1. Introduction

Adhesion is a complex physicochemical phenomenon defined by the ability of substrates to interface and remain bonded [1]. This process results in an adhesive joint characterized by robust contact between materials of similar or dissimilar natures. Such interaction is driven by intermolecular physical forces or covalent chemical bonding at the interface. Currently, the formation of adhesive joints is described by several key theories: mechanical, adsorption, chemical, electrostatic, and diffusion. Table 1 summarizes the fundamental principles of these theories alongside their practical manifestations.

The topic of adhesion remains relevant, as adhesive systems are used both in everyday life (household adhesives, sealants, etc.) and in various industries (medicine, light industry, mechanical engineering and aircraft manufacturing, the space industry, etc.).

In most cases, the term “adhesion” refers to the bonding of solid surfaces using a liquid adhesive. Contact is achieved by good wetting of the surfaces by the adhesive, followed by hardening as a result of a chemical reaction, solvent removal, or a decrease in temperature. This is known as “wet” adhesion. There is also “dry” adhesion, where the formation of an adhesive bond does not involve wetting of the surfaces, and contact between the two solid surfaces is achieved through the microrelief of the surfaces and electrostatic forces [12].

This review focuses on adhesive bonds based on electrostatic attraction and electrostatic adhesion (electroadhesion). Interest in this phenomenon has grown significantly in the past decade, particularly in the context of polymer–polymer systems. Polymers are high-molecular compounds that, due to their unique physical and mechanical properties such as lightness, strength, durability, corrosion resistance, and modifiability, find wide application in various fields, from household packaging to the aerospace industry [13,14,15,16]. However, despite numerous advantages, polymers often face adhesion problems, which can limit their functionality and application [17]. In this context, electroadhesion represents a promising approach for improving the adhesion of polymers to each other and to other materials.

The basic idea of electroadhesion is that when an electric field is applied to two polymer surfaces in contact, an additional force is generated that promotes their attraction (adhesion) [18]. This process is associated with the redistribution of charges on the polymer surfaces, leading to the generation of electrostatic forces that promote adhesion. Importantly, electroadhesion can occur in both static and dynamic conditions, making its study particularly relevant for various technologies, including the assembly and processing of polymeric materials.

Furthermore, electroadhesive systems offer a number of advantages over other types of adhesive systems [19]:

- Adaptability: Electroadhesive systems can be effective for a wide range of materials, from conductors to dielectrics. However, the magnitude of the adhesive force is highly dependent on surface properties (conductivity, roughness) and environmental conditions (humidity, presence of contaminants). For non-conductive substrates, the force is directly proportional to the permittivity, and rough surfaces reduce the effective contact area.
- Low power consumption: Extremely low operating currents of the order of μA are used.
- Simple control system: Easy to configure and adjust.
- Gentle material handling: All adhesion/de-adhesion operations are carried out through electrostatic attraction, eliminating the possibility of mechanical damage.

However, despite their many advantages, electroadhesive systems have two drawbacks: comparatively low bonding strengths compared to other types of adhesive joints and high operating voltages, reaching kV, making the insulation issue particularly pressing. Solving these problems is currently the focus of researchers worldwide, further underscoring the high relevance of this review topic.

For this review, a systematic literature search was conducted in Scopus/Google Scholar/Sciencedirect databases using the following keywords: “electroadhesion”, “polymer–-polymer electroadhesion”, “electroadhesive force”, and “interdigital electrode”. The search covered publications from 2005 to 2025. Earlier classical works were also included. The inclusion criteria were: original experimental or theoretical studies of electroadhesion; availability of quantitative data on electroadhesion force; and use of polymer materials. Works devoted exclusively to general adhesion without an electrostatic contribution were excluded.

## 2. Theoretical Aspects of Electroadhesion

**Figure 1:**
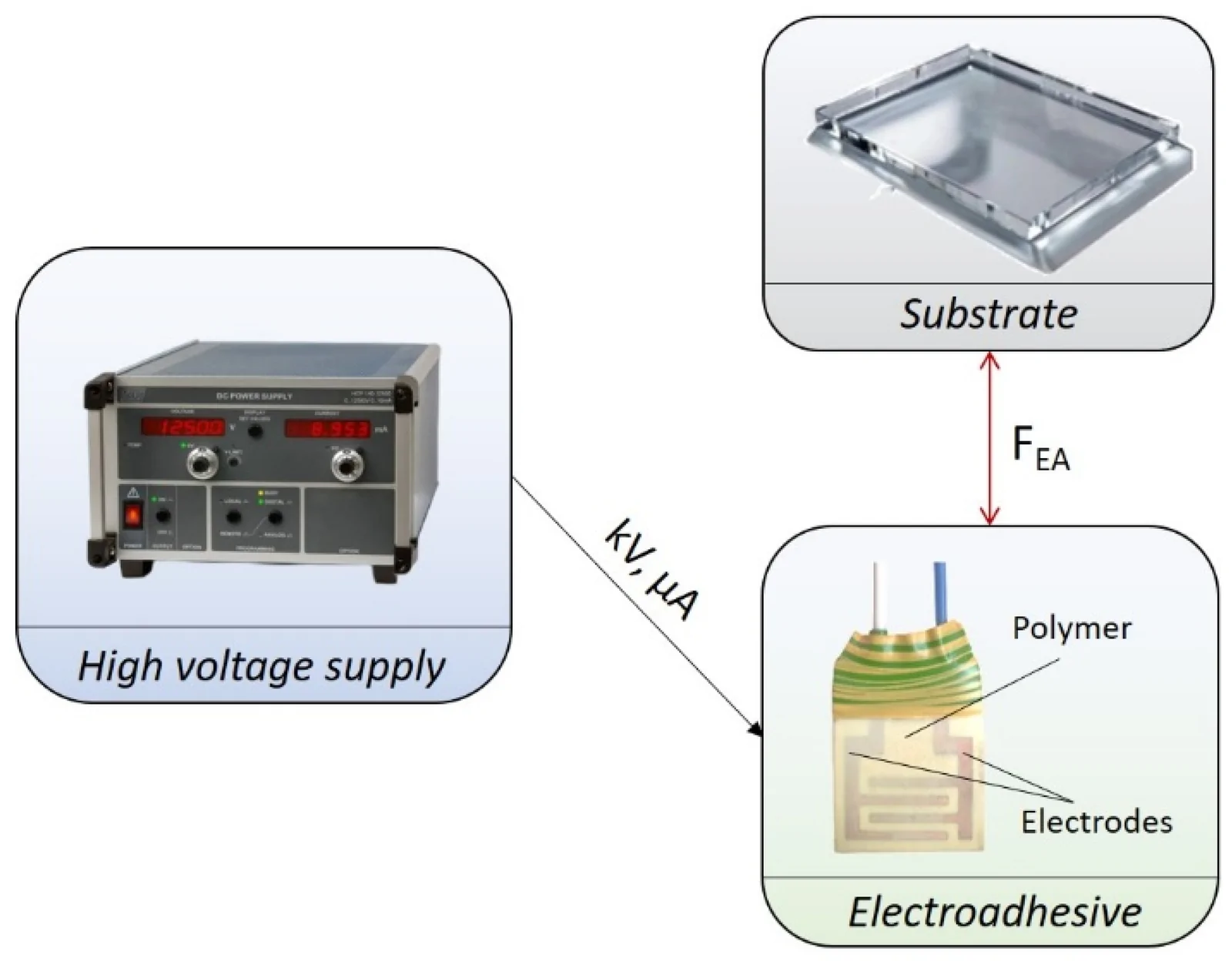
Schematic diagram of the electroadhesive system.

Electroadhesion is a complex dynamic phenomenon in which electrostatic attraction occurs between two charged polymer surfaces in the presence of an external electric field [18]. It is important to note that electroadhesion is not an independent process, but rather a combination of various physical and chemical interactions, including van der Waals forces, dipole–dipole interactions, and ionic bonds. These interactions collectively determine the adhesive strength at the micro- and nanoscale levels, with the contribution of each interaction type depending on the nature of the contacting materials and environmental conditions [20,21]. A typical polymer–polymer electroadhesive system (Figure 1) includes the following components:

- A high-voltage supply;
- An electroadhesive, which consists of electrodes inside a dielectric (polymer);
- A substrate—a dielectric (polymer).

**Figure 2:**
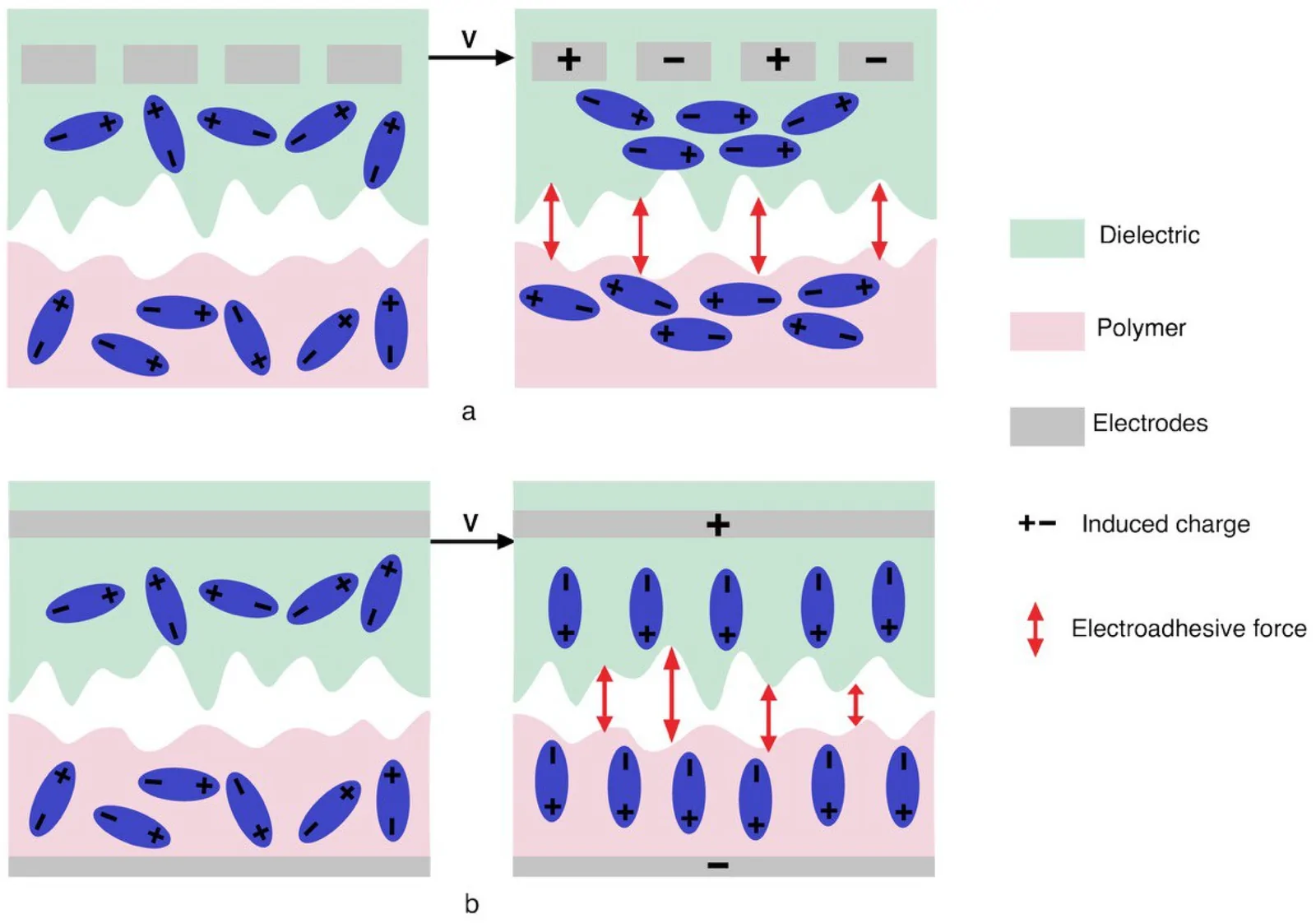
Effect of electrical circuits of electroadhesive systems on dipole orientation: (**a**)—positive and negative poles are embedded in the electroadhesive (interdigital electrode); (**b**)—poles are located on both sides of the dielectric substrates included in the electroadhesive system. See text for details.

A high-voltage supply generates a potential difference between electrodes embedded in a dielectric matrix, which distributes the electric field spatially. In this case, the substrate, which serves as the object of adhesive interaction along with the electroadhesive, acquires induced charges of the opposite sign on its surface. Electroadhesion in polymer–polymer systems is based on the process of electrical polarization of dipoles, which occurs under the influence of an external electric field. The poles of this field can be located either in one substrate of the electroadhesive system (Figure 2a) or in both substrates involved in the electroadhesive contact (Figure 2b). Dipoles arise from the different electronegativity of atoms in macromolecules, creating local charges that are critical for conformational rearrangements and polymer interactions.

When polymer materials are exposed to an electric field, the polymer macromolecules begin to orient in the direction of the field. This leads to a redistribution of charges at the phase boundary. This results in additional interaction forces between the polymer surfaces, leading to increased adhesion [22]. The effectiveness of this process is determined by a combination of factors, among which the key ones are the field strength, the duration of exposure, as well as the dielectric properties and chemical nature of the polymers.

The described effect is based on electrical polarization, the process of generating a net electrical moment within the bulk of a dielectric. Classically, polarization occurs under the influence of an external electric field; however, there are so-called “active” dielectrics (piezoelectrics, pyroelectrics, and photoelectrics), in which polarization can be induced by non-electrical influences. This review focuses on electroadhesive systems based on polymer dielectrics, in which polarization is purely electrical in nature.

The polarization mechanism is discussed in detail in [22,23,24,25]. From the point of view of quantitative characteristics, the resulting electroadhesive forces ($F_{EA}$) are directly proportional to the external electric field strength (E) and the total polarization of the dielectric ($P_{total}$):

(1) $$F_{EA}=EP_{total}$$

(2) $$P_{total}=P_{e}+P_{i}+P_{o}+P_{hop}+P_{if}$$

where $P_{e}$ is the electronic polarization, $P_{i}$ is the ionic polarization, $P_{o}$ is the orientational (dipolar) polarization, $P_{hop}$ is the hopping (thermal) polarization, and $P_{if}$ is the interfacial polarization.

**Figure 3:**
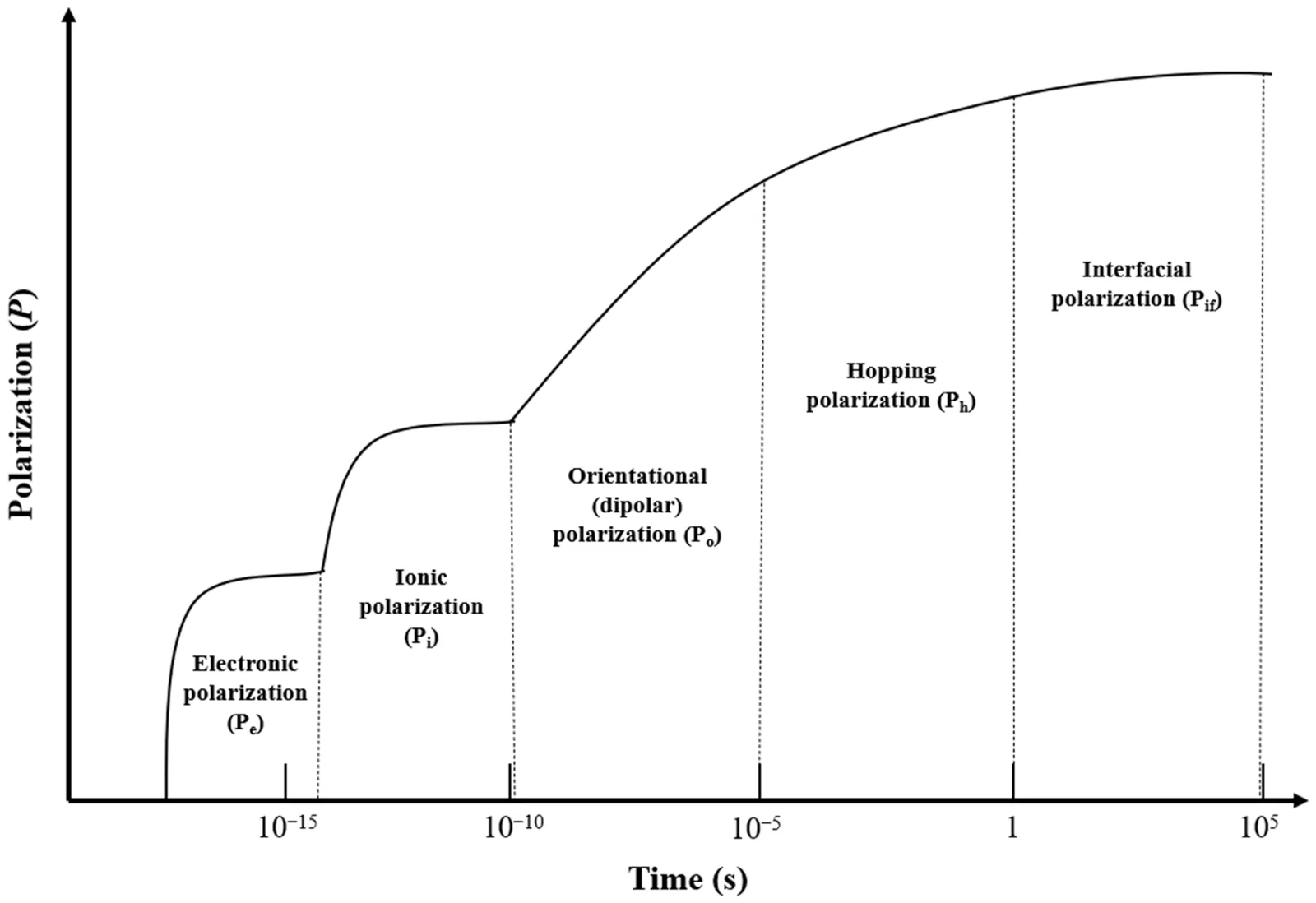
Schematic representation of relaxation times of different types of polarization.

According to experimental data in [18,26,27], each component of the overall polarization has its own relaxation time (Figure 3). The greatest contribution to the overall polarization, and accordingly to the magnitude of electroadhesive forces, is made by orientational polarization, the relaxation time of which is in the range from 10^−10^ to 10^−5^ s and significantly exceeds the relaxation times of electron (~10^−15^ s) and ionic (10^−15^–10^−10^ s) polarizations. To achieve the best adhesive contact in electroadhesive systems, it is recommended to use a constant voltage that promotes the occurrence of the maximum value of electroadhesive forces.

In nature, there are two types of dielectrics: polar and non-polar. Polar dielectric molecules have a permanent dipole moment because the centers of positive and negative charges do not coincide. Polar dielectrics include asymmetric molecules, whose dipole moment increases with the difference in electronegativity of the atoms that make up the molecule [22]. Molecules of non-polar dielectrics do not have a permanent dipole moment because they are symmetrical; that is, they have a common geometric center of positive and negative charges.

**Figure 4:**
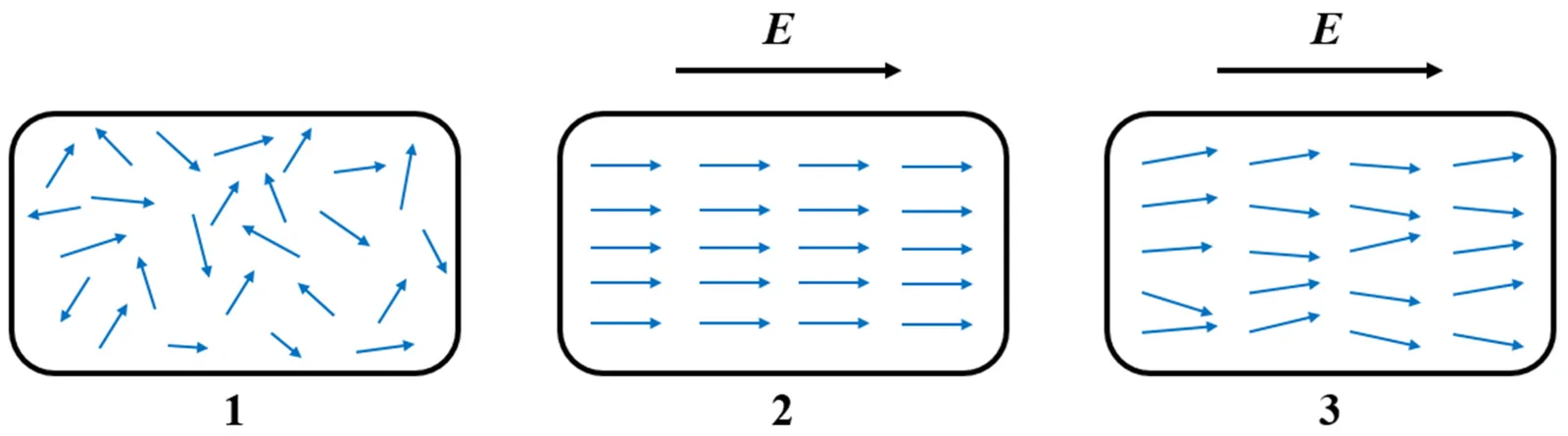
Orientation of permanent dipoles under the influence of an external electric field: 1—in the absence of an electric field; 2—under the influence of an external electric field without taking into account molecular interactions (ideal orientation); 3—under the influence of an external electric field taking into account molecular interactions (real orientation).

Atoms, due to their spherical symmetry, do not possess a permanent dipole moment. However, when exposed to an external electric field, they become polarized, acquiring an induced dipole moment in the direction of the field. This occurs due to the displacement of the electron cloud caused by the applied electric field. When a polar molecule is subjected to an external electric field, it tends to align itself in the direction of the field (Figure 4). However, the continuous thermal motion of the molecules prevents their ideal orientation; that is, increasing temperature hinders the orientation of the dipoles. However, the stronger the external electric field, the closer the dipoles come to their ideal orientation.

Under an external electric field, polar molecules and atoms behave as dipoles, aligning along the field’s direction. However, interactions between permanent and induced dipoles disrupt their ideal orientation. The resulting polarization leads to charge separation in the material: a net positive charge accumulates on one side, while a net negative charge accumulates on the opposite side.

**Figure 5:**
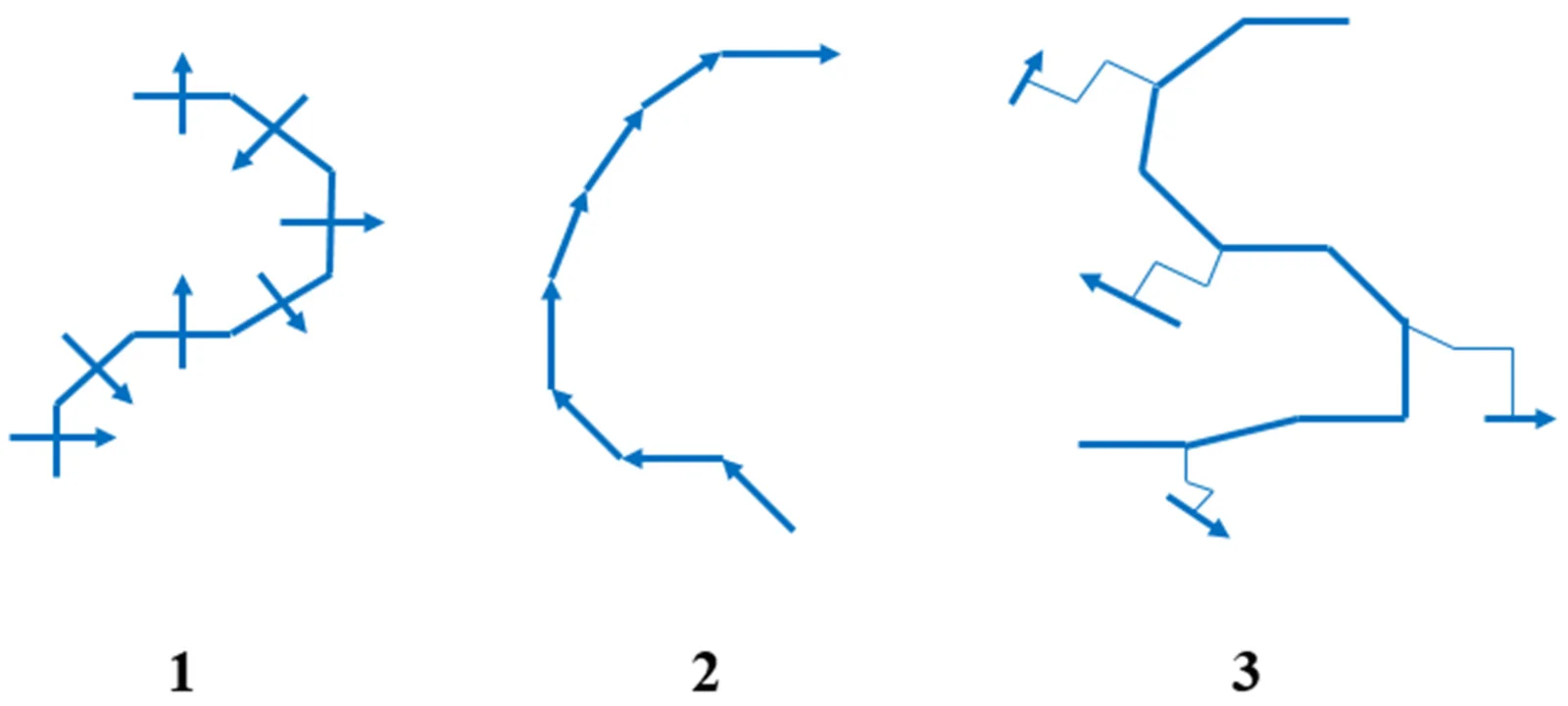
Examples of possible configurations of permanent dipoles relative to polymer chains: 1—dipoles dividing the links in half (across the chain links); 2—dipoles located parallel to the chain links; 3—dipoles that are side groups of the polymer chain.

The occurrence of orientational polarization in polar polymers is associated with the mobility of permanent dipoles. The ability of these dipoles to align parallel to the field is determined by the polymer structure (network, branched, linear). Furthermore, their local environment influences this: a dipole associated with a flexible polymer chain is more mobile and more easily oriented along the applied electric field than a dipole associated with a rigid polymer chain. Examples of possible configurations of permanent dipoles relative to polymer chains are shown in Figure 5. Importantly, in the amorphous phase, dipoles are less constrained and have greater mobility than in the crystalline phase. This allows them to more easily orient themselves relative to the applied electric field.

Thus, the strength of the adhesive bond in electroadhesive polymer–polymer systems is influenced by both the characteristics of the electric field and the properties of the used polymers. To achieve maximum electroadhesive strength without the need to increase voltage, amorphous polar polymer materials with highly electronegative atoms in the polymer chain can be used.

## 3. Modeling of Electroadhesion Effect

**Figure 6:**
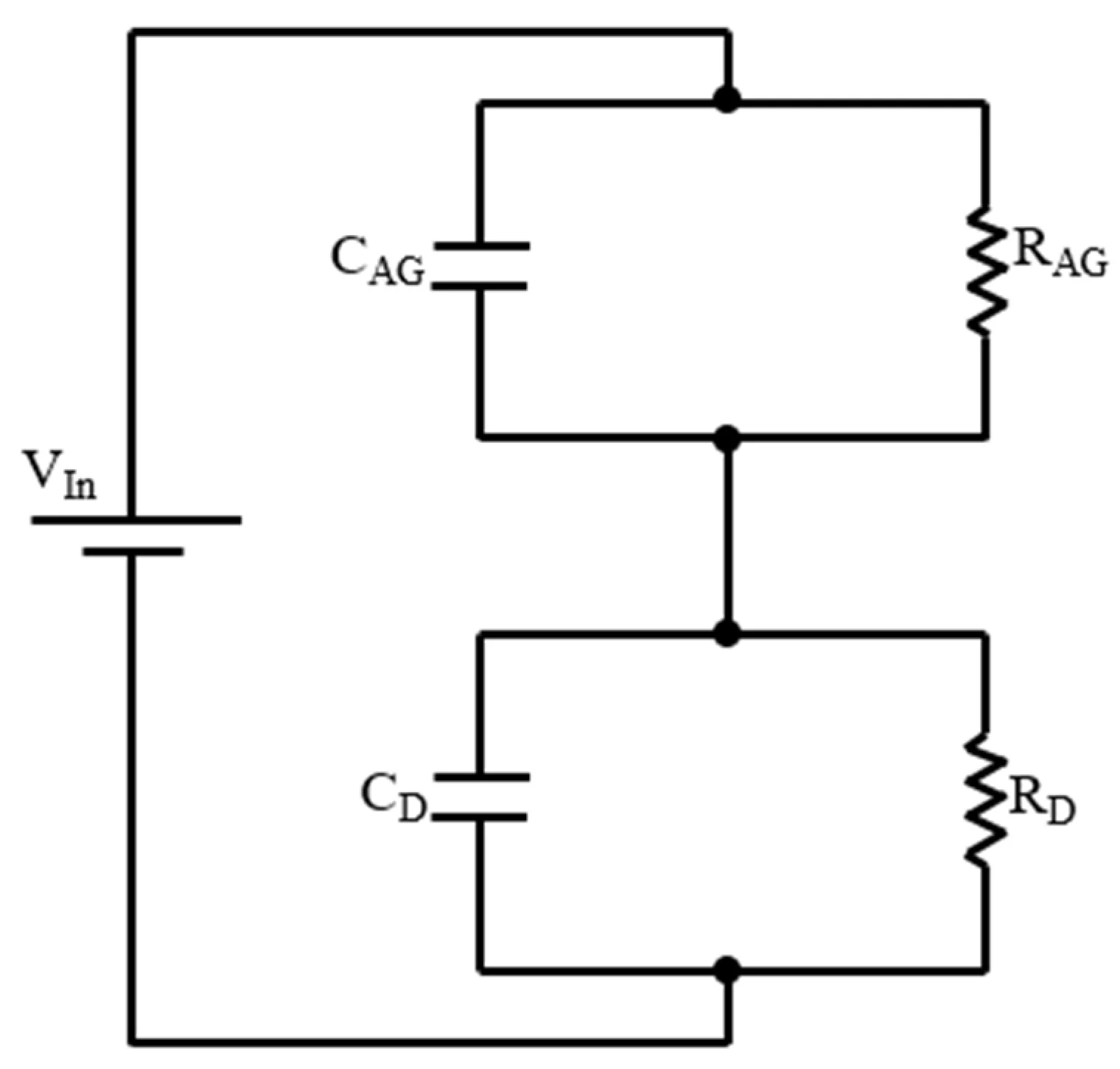
Johnsen–Rahbek electroadhesion model: R_AG_, C_AG_—air gap resistance and capacitance elements; R_D_, C_D_—dielectric resistance and capacitance elements; V_In_—input voltage.

To minimize the resources and time required for experimental verification of the electroadhesive effect, modeling is used at the initial stage. The classical Johnsen–Rahbek model (Figure 6) is sometimes used [28,29,30,31,32], which interprets the electroadhesive interaction through an equivalent electrical circuit.

This model represents the system as two series-connected parallel circuits with their own capacitance and resistance, as discussed in detail in [28,29,30,31,32]. For simplicity, one circuit combines the behavior of the electroadhesive and the substrate, while the second describes the behavior of the air gap.

**Figure 7:**
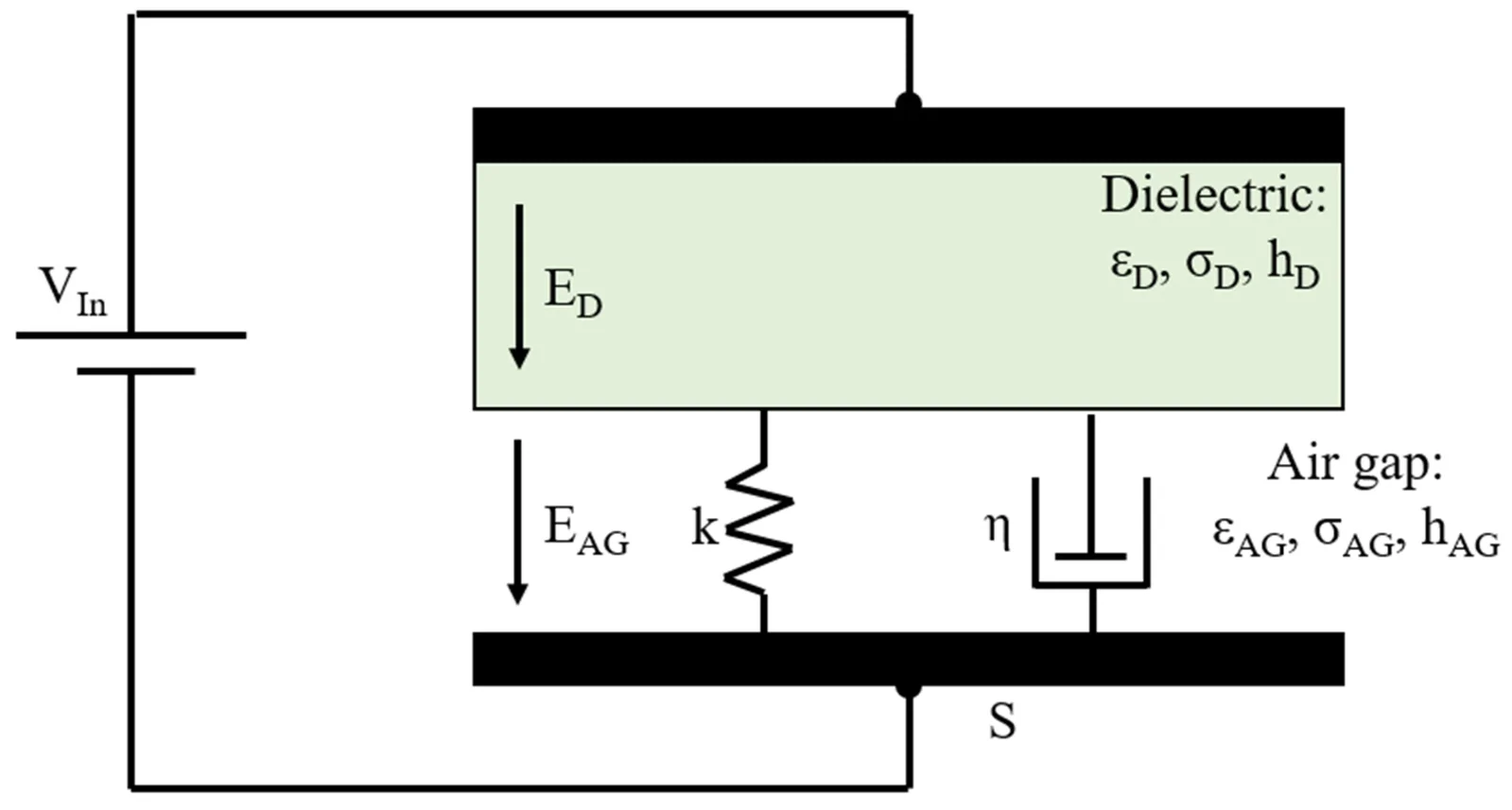
Nakamura and Yamamoto electroadhesion model: ε_D_, σ_D_, h_D_—permittivity, electrical conductivity and thickness of the dielectric; ε_AG_, σ_AG_, h_AG_—permittivity, electrical conductivity and thickness of the air gap; E_D_, E_AG_—electric field strengths in the dielectric and air gap, respectively; V_In_—input voltage; S—electrode area; k—stiffness coefficient; η—damping coefficient.

At the same time, Nakamura and Yamamoto [33] developed an electromechanical model for a more detailed description of the electroadhesive effect (Figure 7), taking into account both the electrical and mechanical aspects of the process. Their work aims to address the limitations of previous models, in particular the Johnsen–Rahbek model, which does not account for the mechanical behavior of the electrodes and the dynamics of force changes under constant voltage.

The model (Figure 7) simultaneously describes the redistribution of the electric field and charges in a multilayer structure and changes in contact geometry (gap thickness, actual contact area, and microroughness). The key difference in their approach is that the electroadhesive force is considered not as a statically calculated value for given parameters, but as the result of interrelated dynamics: an applied DC voltage causes charge accumulation, which changes the effective field in the gap and dielectrics; simultaneously, the mechanical component describes the compliance/rigidity of the electrodes and clamping elements, causing the contact to “pull up”. The gap decreases, the actual contact area increases, and, consequently, the equivalent electrical parameters (capacitance and resistance) change.

Thus, the main differences between the Nakamura and Yamamoto model and the Johnsen–Rahbek model are the presence of a mechanical component in the form of spring and piston elements, as well as the use of electric field strength E instead of voltage V to more easily describe the relationship between the mechanical and electrical components of the model. For each model, the electroadhesive force is calculated using Maxwell’s equation. It is important to note that the Johnsen–Rahbek model is simple and convenient for quickly estimating force in systems with rigid contact. However, it does not account for the mechanical deformation of polymer materials, which is especially important for soft and elastic polymers. The Nakamura and Yamamoto model, in turn, takes into account dynamics and mechanical behavior but requires viscoelastic parameters, which are difficult to determine experimentally for many polymers. For polymer–polymer systems, the most promising approach is a combination of the Maxwell stress tensor, taking into account boundary conditions, and data on the dielectric relaxation of polymers. This approach allows for high-quality force predictions without labor-intensive experiments. A summary of the differences between the two models is presented in Table 2.

Currently, in the field of electroadhesive modeling, special attention is paid to studies that allow selection of the optimal geometry of built-in electrodes [18,26,27,34,35,36,37,38,39,40] to achieve the greatest electroadhesive forces, as well as to calculate the expected electroadhesive forces under various conditions [20,21,29,41,42,43,44,45,46,47,48]. Several electrode design variations can be found in the literature (Table 3), differing in geometry, placement method, and functional features [18,26,27,34,35,36,37,38,39,40]. The circuit illustrated in Figure 2a, where both poles are located within a single substrate of the electroadhesive system, is implemented using interdigital electrodes or concentric circle electrodes. Electrodes of the other types presented in Table 3 are positioned according to the circuit shown in Figure 2b on both sides of the contacting dielectric substrates of the electroadhesive system.

**Figure 8:**
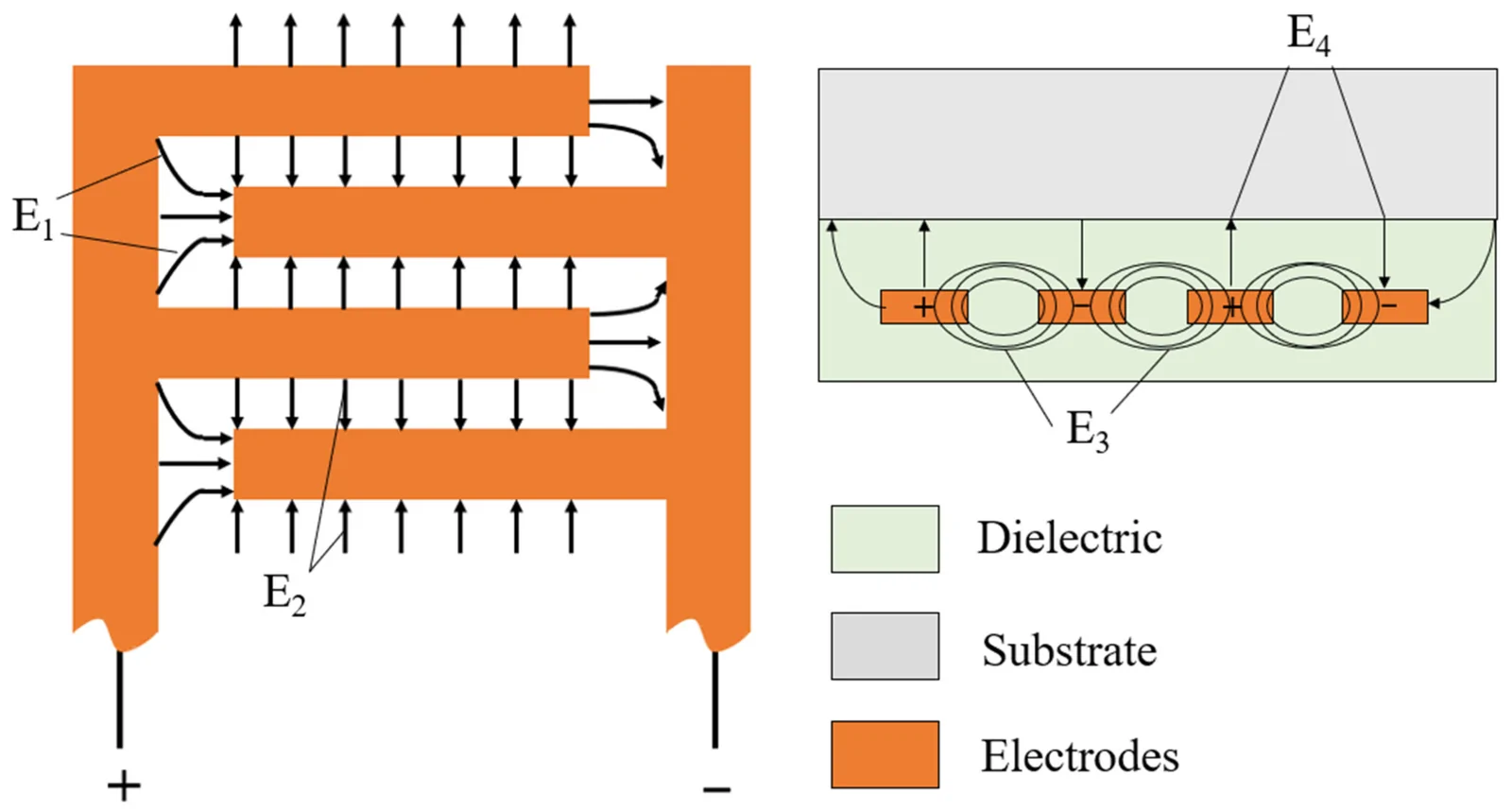
Propagation of electric fields in the electroadhesive system: E_1_—fringing fields; E_2_—fields between electrodes due to electrode thickness; E_3_—fields between the coplanar electrode surfaces; E_4_—fields between the electrodes and the substrate. The arrows show the lines of electric field strength.

Of all the electrode types considered, the interdigital design is the most commonly used. This is due to the electric field’s unique propagation, allowing it to penetrate deeper into the dielectric layer and create significant electroadhesive forces [18]. A schematic of the field propagation in interdigital electrodes is shown in Figure 8.

**Figure 9:**
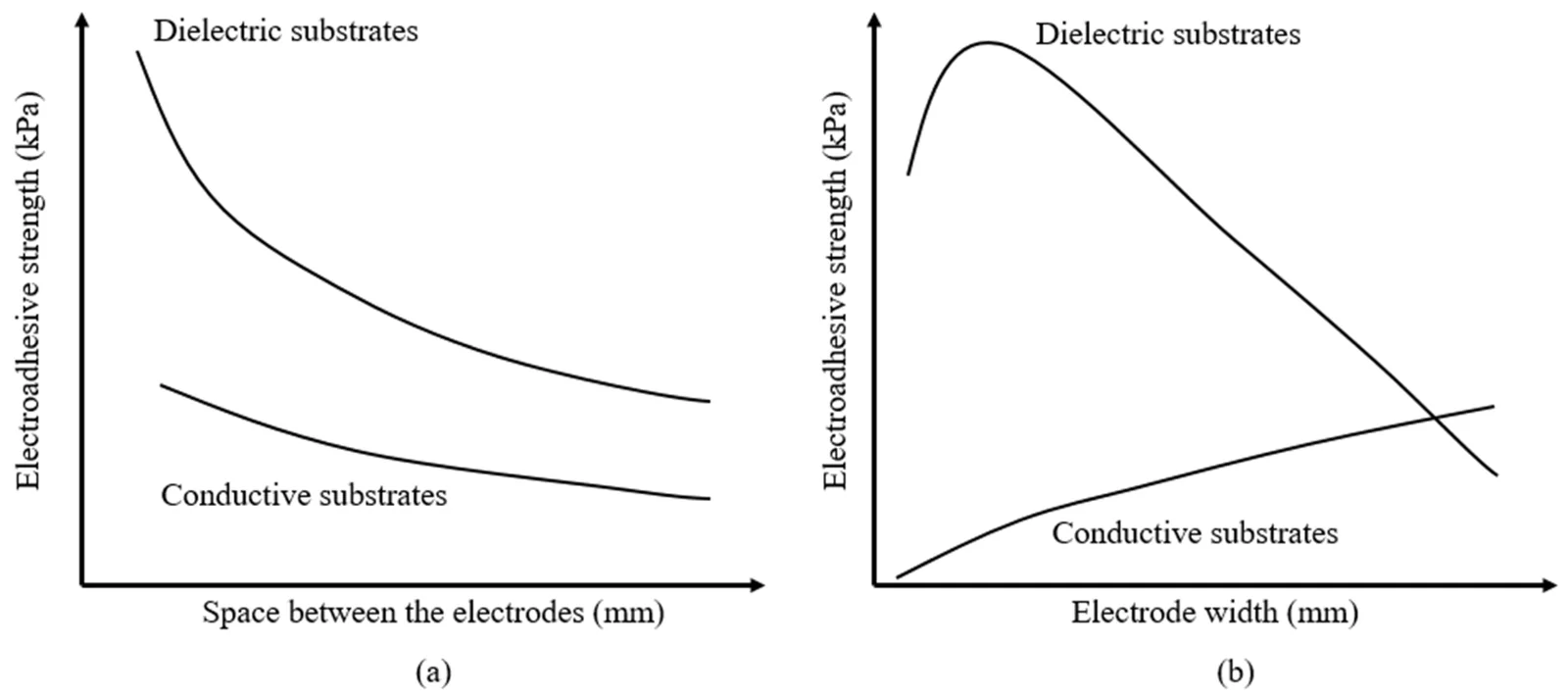
General view of the dependence of the electroadhesive strength on the geometric parameters of interdigital electrodes: (**a**)—distance between electrodes; (**b**)—width of electrodes.

After the optimal electrode geometry was determined, research focused on its effect on the strength of electroadhesion [18,26,37,44,45]. Numerous experiments revealed relationships linking electroadhesion strength to geometric parameters such as electrode width and distance between them. These relationships are presented in general terms in Figure 9.

It is important to note that when studying electroadhesion, the type of substrate with which the electroadhesive comes into contact must be taken into account. For conductive substrates, the electroadhesive force is directly proportional to the electrode width, while for dielectric substrates, there is an “optimum” after which further increases in electrode width lead to a decrease in electroadhesive forces.

The final stage of modeling the electroadhesive effect is the calculation of the expected electroadhesive forces. The processes for deriving the final equations and some results of numerical experiments are described in detail in [21,28,29,30,31,32,34,41,42]. The force of electroadhesion upon contact with substrates of any nature (conductors, semiconductors, dielectrics) is determined by the integral of the Maxwell stress tensor $T_{ij}$ (ignoring the influence of magnetism) over the contact surface. For a qualitative assessment in a homogeneous dielectric, a simplified Equation (1) can be used. This approximation is valid only under the condition that the electric field and polarization vector are codirectional, and edge effects can be neglected. For heterogeneous systems, numerical modeling may be required. The general form of the Maxwell stress tensor is presented in Equation (3).

(3) $$T_{ij}=\varepsilon_{0}\left(E_{i}E_{j}-\frac{1}{2}\delta_{ij}E^{2}\right)$$

where $\varepsilon_{0}$ is the permittivity of vacuum; *E* is the electric field strength; *δ* is the Kronecker delta.

The essence of modeling the electroadhesive effect lies in selecting the optimal electrode model and design for given conditions. By selecting the required model and geometry, it becomes possible to more reliably predict the behavior of electroadhesive polymer–polymer systems using CAE software packages (COMSOL Multiphysics 6.4).

## 4. Electroadhesive Manufacturing Technologies

Modern electroadhesives are composite structures consisting of conductive electrodes placed on a polymer substrate and insulated by a dielectric layer. Manufacturing technologies for these electroadhesives are rapidly developing, but they face limitations such as:

- Limitations of existing processing methods;
- Diversity of materials;
- Exposure to external factors;
- Difficulty in scaling.

**Figure 10:**
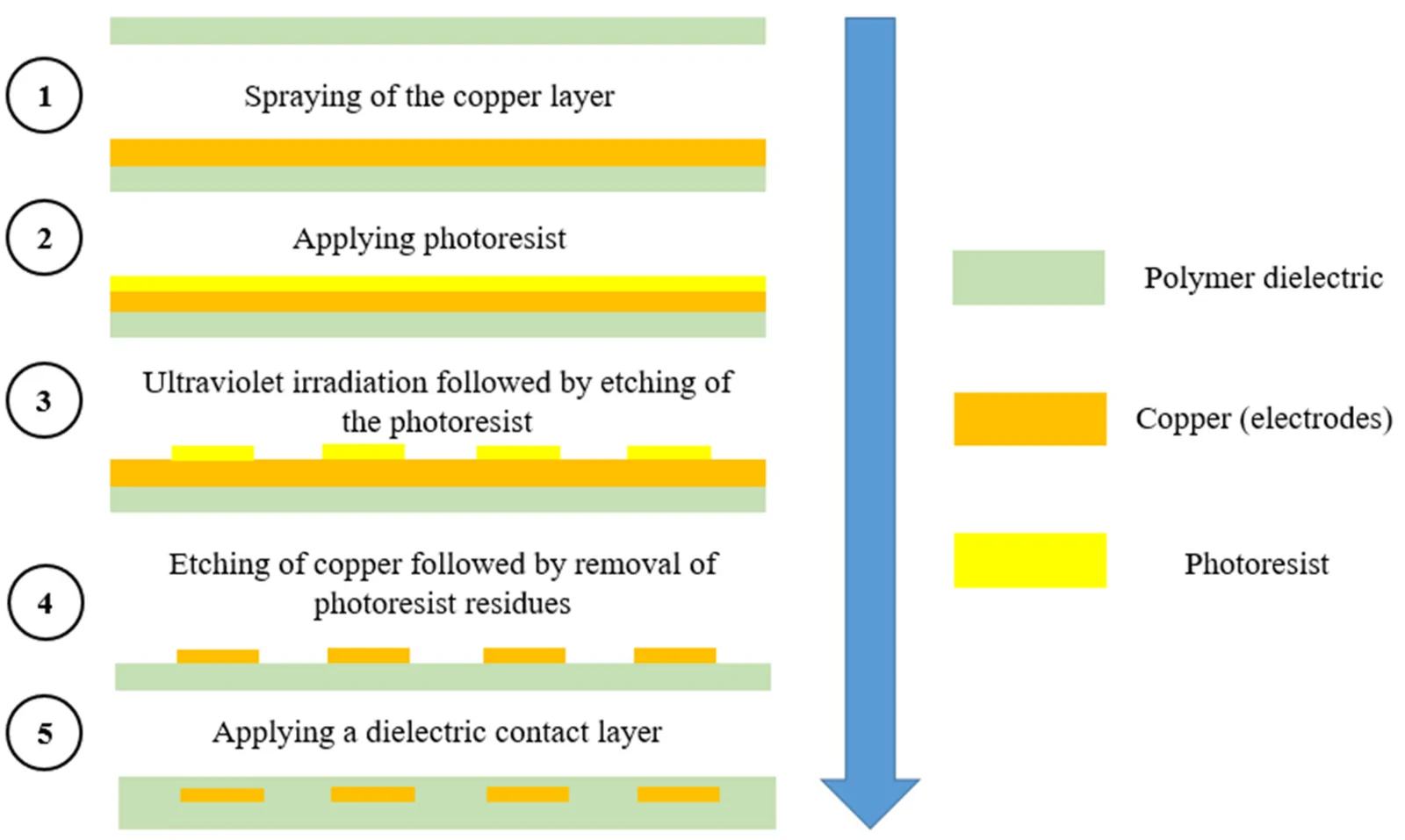
Manufacturing technology of electroadhesives.

Currently, the main technology for the production of such electroadhesives is surface micromachining (Figure 10) [34].

This technology is based on photolithographic processes, which include the following steps [19,34,49]:

(1) Spraying of a metal layer on a polymer substrate (electrodeposition, vacuum deposition, plasma deposition);
(2) Removal of oxide and grease films from the metal surface (acid cleaning), followed by application of photoresist (by centrifugation) and drying in a heat chamber at a temperature of 100–120 °C;
(3) Exposure and development of the photoresist: irradiation of the photoresist layer with ultraviolet radiation through a photomask with a given pattern, followed by its development, i.e., removal of the irradiated area in the case of positive photoresist, or removal of the unirradiated area if negative photoresist was used;
(4) Etching (plasma) followed by removal of residual photoresist;
(5) Applying a dielectric contact layer.

In cases where the electrode has a simple shape (a solid rectangle or circle), the manufacturing technology is not complicated by photolithographic operations, but is limited to layer-by-layer deposition of materials [50].

There are also studies aimed at simplifying the production of electroadhesives and abandoning photolithography in favor of mechanical methods. For example, in [51], the authors used a CNC machine to cut an electrode from foil-clad fiberglass, followed by filling it with a dielectric (polymer) in a silicone mold. Another group of scientists [52] completely 3D-printed an electroadhesive, using a conductive carbon-filled filament for printing the electrode.

## 5. Factors Influencing Electroadhesive Interactions

**Figure 11:**
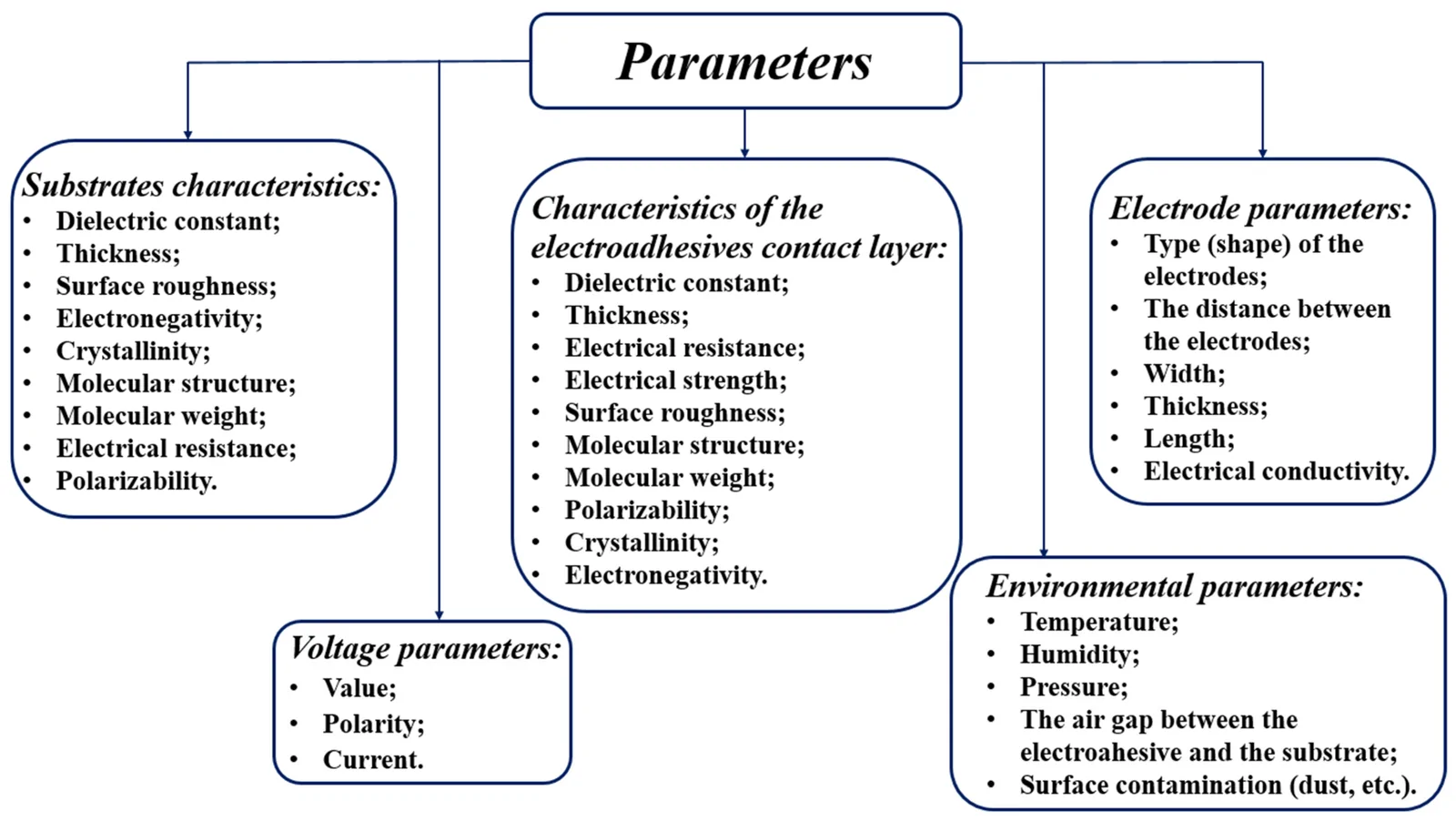
Factors influencing electroadhesive forces.

Electroadhesion is an extremely sensitive phenomenon. Electroadhesive forces are influenced by a large number of factors, including voltage parameters, characteristics of the substrate and dielectric contact layer of the electroadhesive, electrode parameters, and the surrounding environment [19]. A more detailed description of the parameters influencing electroadhesive interactions is presented in Figure 11.

**Figure 12:**
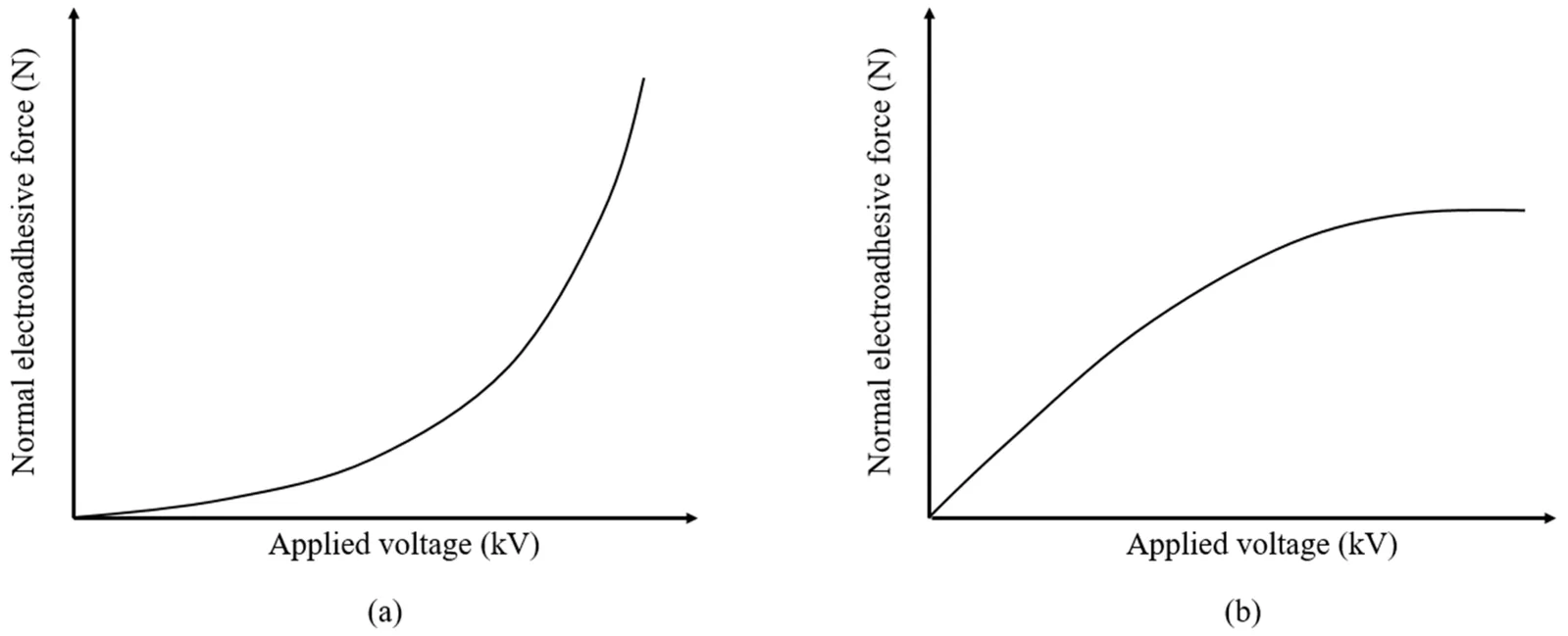
General view of the dependence of electroadhesion forces on the applied voltage: (**a**) theoretical calculation; (**b**) real systems.

The works [18,35,37,51,53,54] describe in detail how the electroadhesive effect behaves depending on the applied voltage. As the voltage increases, so do the electroadhesive forces. According to theoretical calculations, the electroadhesive forces should increase exponentially, reaching their maximum only at the moment of electrical breakdown. However, real electroadhesive systems have shown a different relationship. Upon reaching a certain voltage level, called the “maximum”, the electroadhesive forces reach their peak, after which they plateau. This means that at a certain voltage, the effect of “saturation” of electroadhesion is achieved (Figure 12) [28,51,54].

The magnitude of the maximum possible electroadhesive forces in this case is determined by the dielectric properties of the substrate and the contact layer of the electroadhesive, primarily their dielectric constant [18,37,51,54].

**Figure 13:**
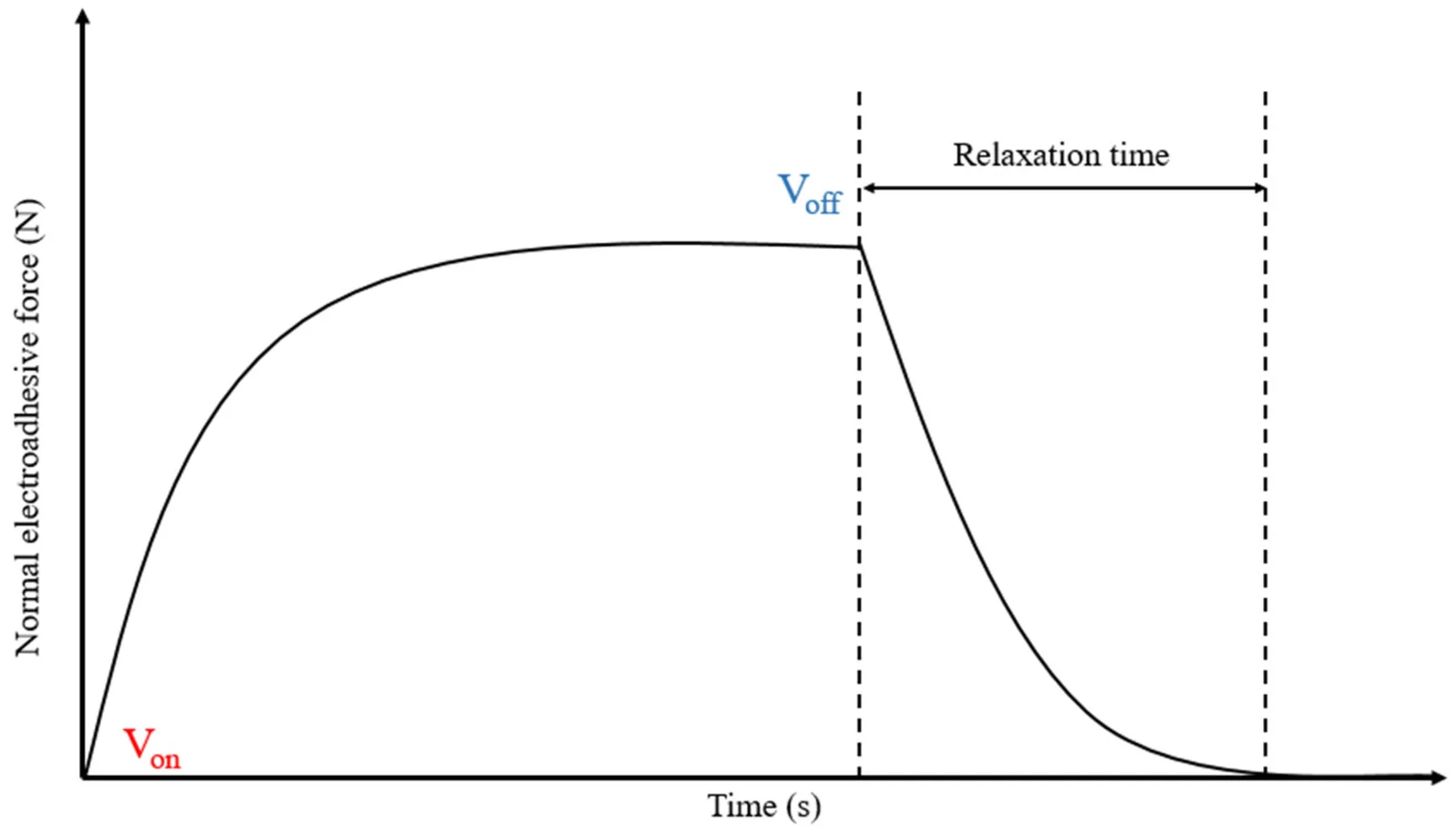
Typical relaxation curve of electroadhesion.

Although constant voltage is typically used in electroadhesive systems, there are also studies [29,33,55] that investigate the phenomenon of electroadhesion under alternating voltage. This issue arose primarily due to the relaxing nature of the electroadhesive effect (Figure 13), which, in some cases, increased the adhesion–deadhesion cycle time in electroadhesive systems.

**Figure 14:**
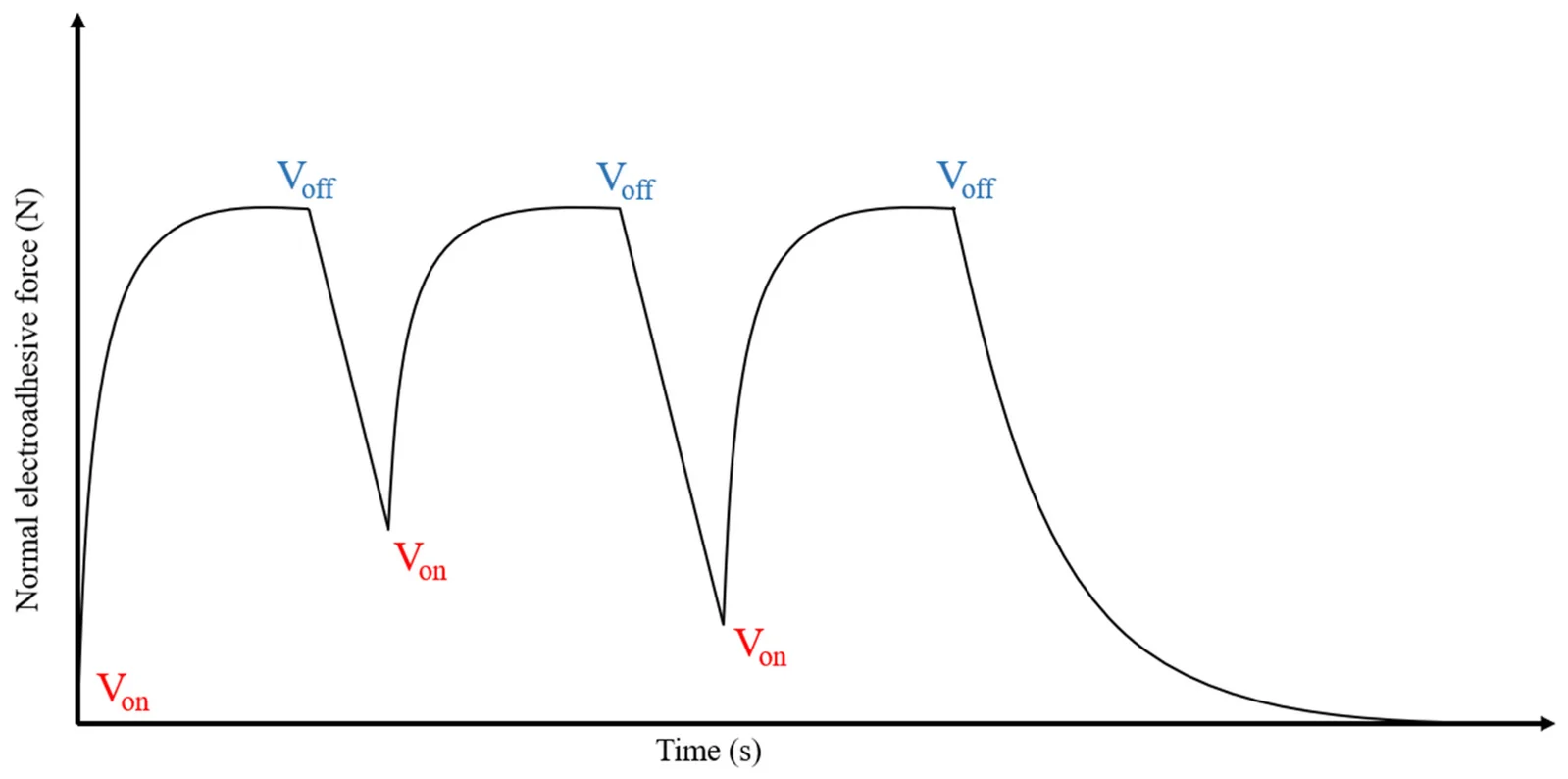
General view of the charge–discharge diagram.

The electroadhesive forces achieved with alternating voltage are slightly lower than those achievable with constant voltage. However, the use of alternating voltage minimizes the influence of the relaxation nature of electroadhesion and reduces the adhesion–deadhesion cycle time, which is extremely important in some cases [32,33,38,51]. A description of the relaxation nature of electroadhesion at constant voltage, as well as stability during adhesion–deadhesion cycles, is presented in [51] using the example of charge–discharge (adhesion–deadhesion) diagrams, the general view of which is shown in Figure 14. In this work, the charging time was constant and amounted to 30 s, while the discharge time varied from 10 to 60 s to clearly demonstrate the relaxation of electroadhesive forces. It was noted that for various systems, 10 s is insufficient for complete relaxation of electroadhesion, while after 30 s of discharge, residual forces of electroadhesive interactions were not recorded. Also, the results presented in [51] enable us to speak with cautious optimism about the stability of electroadhesive systems, since throughout all adhesion–deadhesion cycles, the maximum value of electroadhesive forces was achieved after 10 s and remained unchanged until the start of discharge.

**Figure 15:**
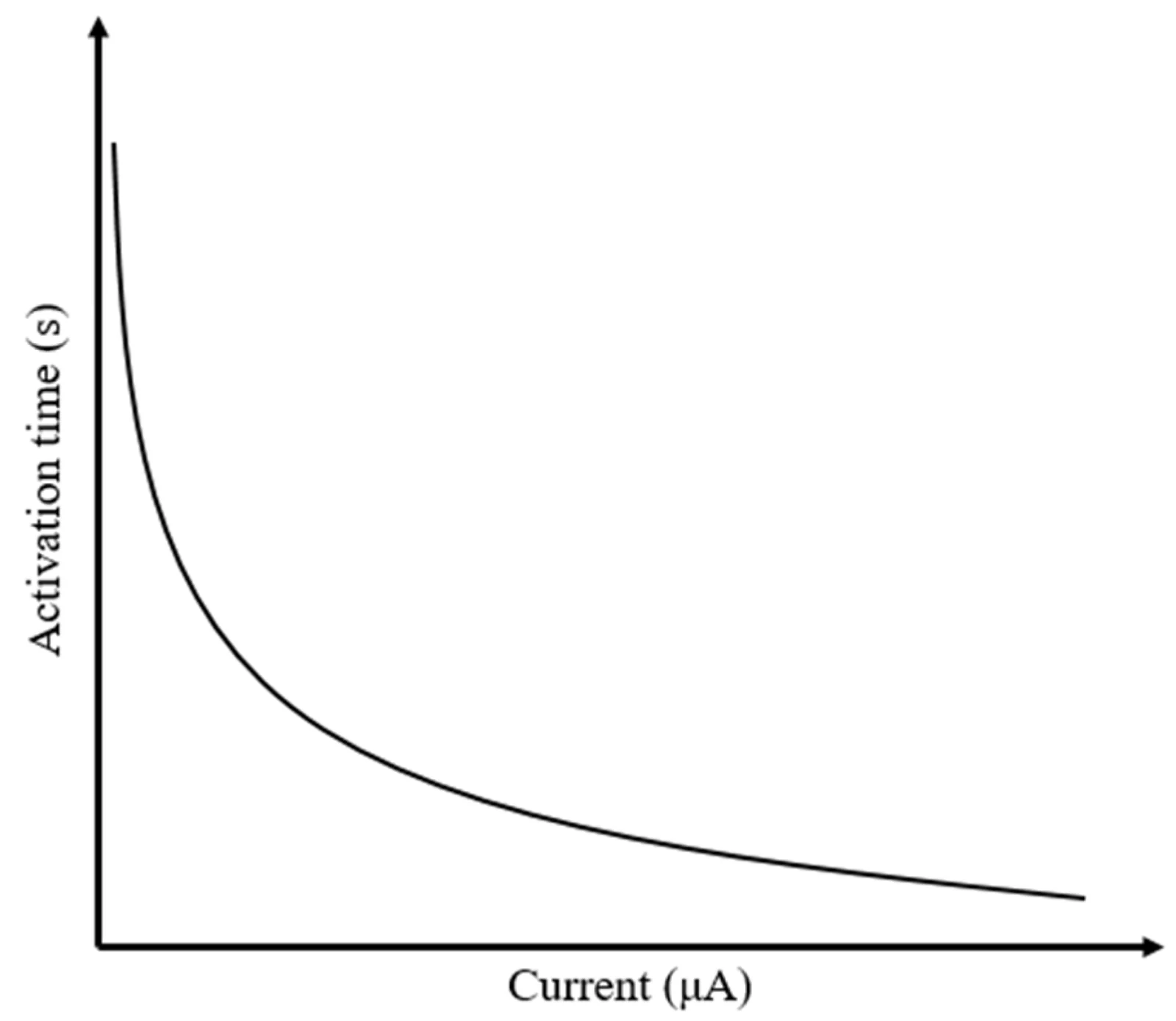
Typical dependence of the activation time of electroadhesion on the current strength.

Work [51] showed that current strength influences the activation time of electroadhesion, i.e., the time it takes to reach maximum electroadhesive forces in the system. The dependence has the form shown in Figure 15.

**Figure 16:**
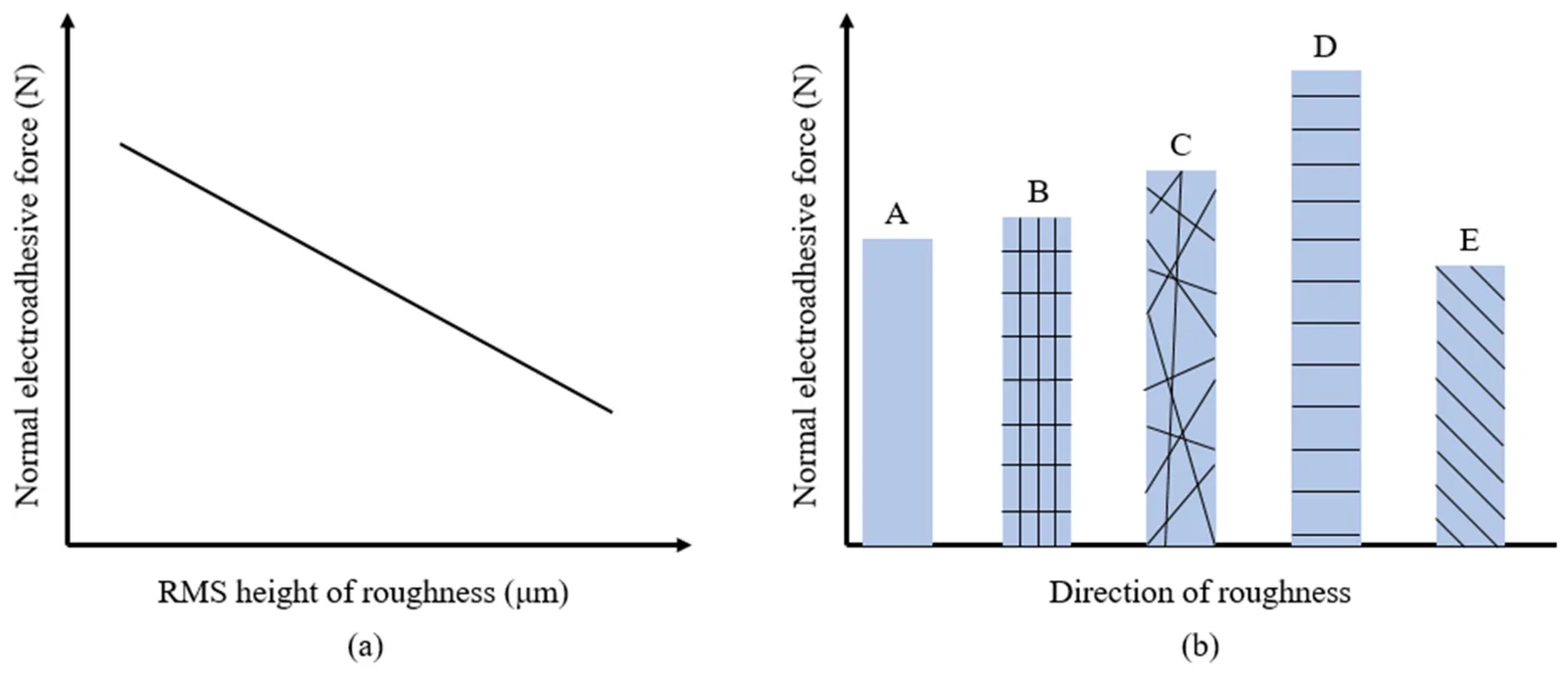
(**a**) General trend of the influence of the RMS height roughness parameter on the electroadhesive forces; (**b**) Influence of the roughness direction on the electroadhesive forces: A—initial substrate; B—bidirectional roughness; C—random roughness; D—horizontally directed roughness (in this case, the roughness direction is across the direction of the electric field lines); E—the roughness is directed at an angle of 45°.

Another parameter that significantly affects the electroadhesive forces is the surface condition of both the substrate and the contact layer. This includes the surface texture (smooth or rough), as well as the presence of foreign inclusions (contamination). In [19,54,56], the influence of the roughness direction on the electroadhesive forces was studied in detail, as well as the influence of such a roughness parameter as the root-mean-square height S_q_ (RMS height). Summarizing the obtained results, it can be concluded that the roughness direction (horizontal, bidirectional, random, at an angle of 45°, vertical, etc.) has a significant effect on the difference in the generated electroadhesive forces. It should be noted that in all the analyzed studies the influence of the roughness direction relative to the electric field lines in the system was studied. It was found that the greatest electroadhesive forces arise when the roughness direction coincides with the direction of the electric field lines. The influence of the S_q_ parameter on the electroadhesive forces was noted: the generated electroadhesive forces are inversely proportional to the RMS height value, that is, with a decrease in the S_q_ parameter, the magnitude of electroadhesive interactions increases. However, the authors of [19,54] note the fact that a change in the electroadhesive forces is observed only when the difference between the S_q_ parameters of two substrates is more than 2 μm. General trends in the influence of the roughness direction and the S_q_ parameter are presented in Figure 16.

**Figure 17:**
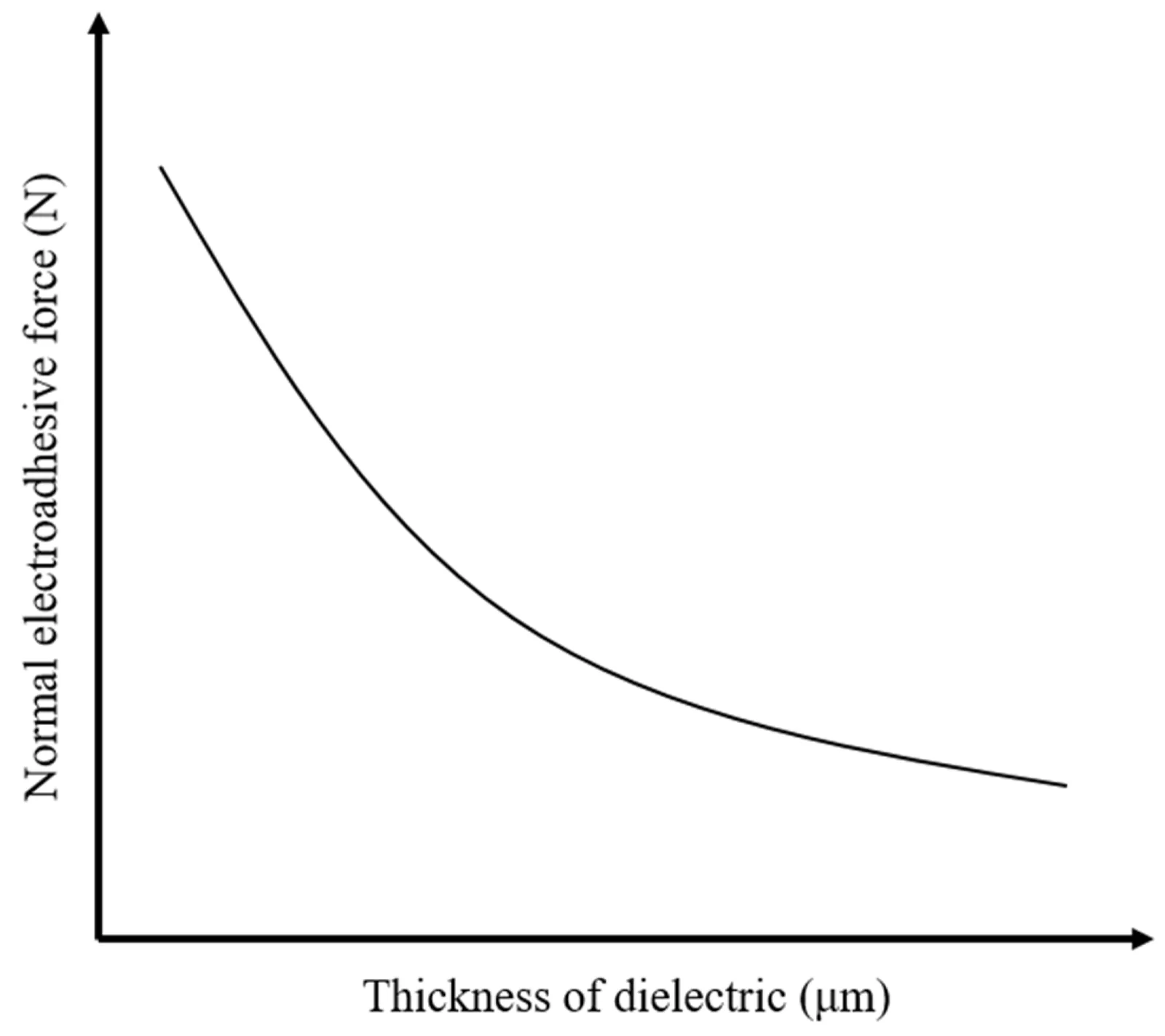
Typical curve of the electroadhesive force depending on the thickness of the dielectric of the contact layer of the electroadhesive.

The influence of material characteristics on the generated electroadhesive forces, a list of which is presented earlier in Figure 11, is described in [18,35,38,51,54,57,58,59]. Data on the influence of the dielectric contact layer thickness on electroadhesive interactions are presented in [18,35]. Electroadhesive forces are inversely proportional to the contact layer thickness; i.e., the greater the thickness of the dielectric layer of the electroadhesive, the lower the generated electroadhesive forces. A typical curve of electroadhesive forces as a function of the contact layer thickness is shown in Figure 17.

**Figure 18:**
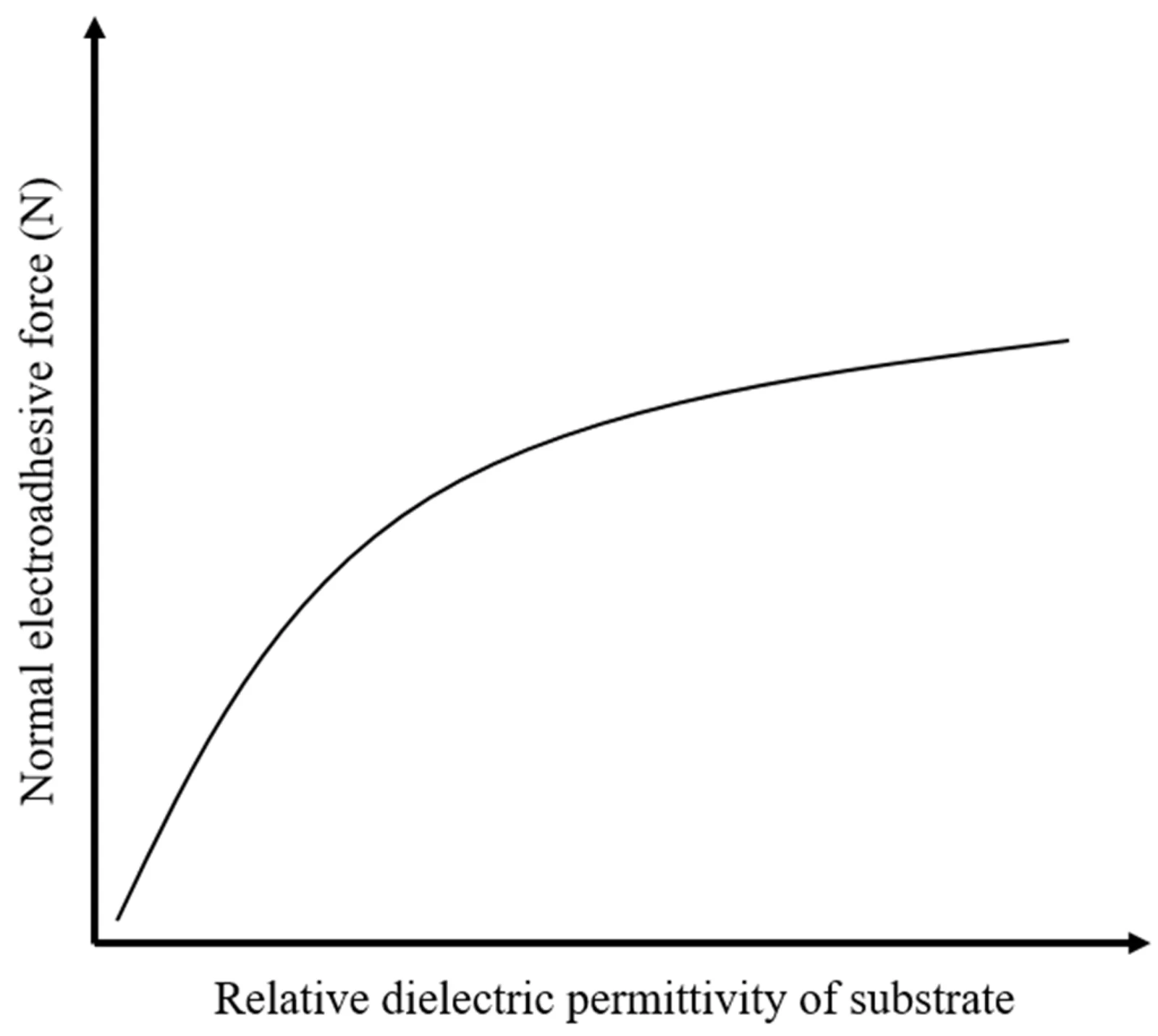
Typical curve of dependence of electroadhesive forces on relative permittivity.

In [38,51,54,57,58,59], the occurrence of the electroadhesive effect and the strength of the electroadhesive forces arising upon contact of an electroadhesive with materials of varying electrical conductivity, including conductors, semiconductors, and dielectrics, were thoroughly studied. Particular attention was paid to the interaction with dielectrics with different permittivity. Analysis of the experimental data allowed us to draw an unambiguous conclusion: the greatest electroadhesive forces arise upon contact of an electroadhesive with conductors, i.e., with metals. In the case of polymer–polymer systems, the electroadhesive forces are directly proportional to the permittivity of the material with which the electroadhesive is in contact. A typical curve of the dependence of electroadhesive forces on the dielectric constant is shown in Figure 18.

In our published work [60], the influence of polymer polarity on the magnitude of electroadhesive interactions, expressed through electroadhesive strength, is described. The influence of polarity is demonstrated using the example of an ethylene-vinyl acetate copolymer with different vinyl acetate contents (7, 20, 28, and 40 wt.%), where the extreme points were low-density polyethylene (non-polar) and polyvinyl acetate (polar). With an increase in polymer polarity, a gradual increase in electroadhesive strength was observed from 6 Pa for non-polar polyethylene to 41 Pa for polar polyvinyl acetate.

In addition to the influence of the polymer polarity on electroadhesive interactions, the influence of the degree of crystallinity on the magnitude of electroadhesive strength was noted in [60]. The change in the value of electroadhesive strength was demonstrated using the example of EVA28 films (EVA with 28 wt.% vinyl acetate) with a degree of crystallinity of 11% and 15%. The degree of crystallinity of the EVA28 film was increased by the orientation drawing method. The value of electroadhesive strength for samples with a degree of crystallinity of 11% and 15% was 46 Pa and 38 Pa, respectively. A decrease in the value of electroadhesive interactions with an increase in the degree of crystallinity is explained by a decrease in the mobility of molecular dipoles. As a result, the orientation of dipoles under the influence of an external electric field is hindered, which worsens electroadhesive interactions and, as a consequence, reduces the value of electroadhesive strength.

**Figure 19:**
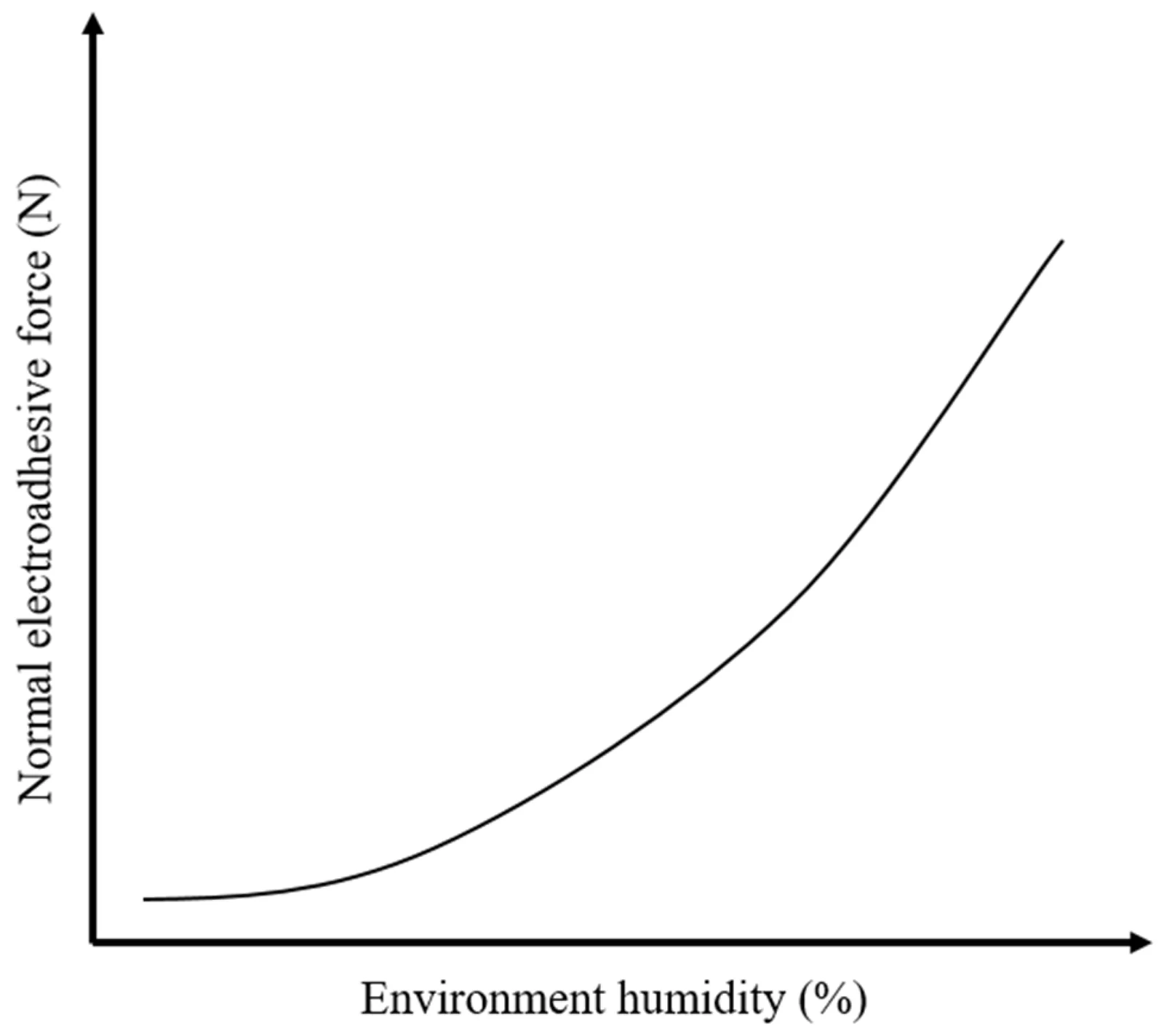
Typical curve of the influence of environmental humidity on the generated electroadhesive forces.

In [18,54,58,59,61,62], the influence of atmospheric conditions on electroadhesive forces was studied. Varying temperature and pressure in narrow ranges from 21 to 25 °C and from 975 to 1025 hPa did not reveal any effect on electroadhesive forces. Changing the humidity value in a wide range from 35 to 65% made it possible to reveal its significant influence on electroadhesive interactions [18,54,58,61,62]. Experimental data presented in [18,54,58,62] confirm that electroadhesive forces increase with increasing humidity. This is apparently due to the increased concentration of polar water molecules on the surface, where they play two important roles: they ensure closer contact between the surfaces of the electroadhesive and the substrate, and they actively respond to the applied electric field, significantly contributing to electroadhesive interactions. However, the effect of humidity on electroadhesive interactions is multifactorial. In addition to increasing the concentration of polar water molecules, an adsorbed water layer can reduce the contact resistance between the electroadhesive and the substrate, facilitating charge transfer and increasing the effective contact area. High humidity can also cause a continuous water film to form, which can increase leakage currents and create a shielding effect, reducing the electric field strength at the interface. Thus, the influence of humidity depends on the balance between positive and negative effects. A typical curve for the effect of humidity on electroadhesive forces is shown below in Figure 19.

**Figure 20:**
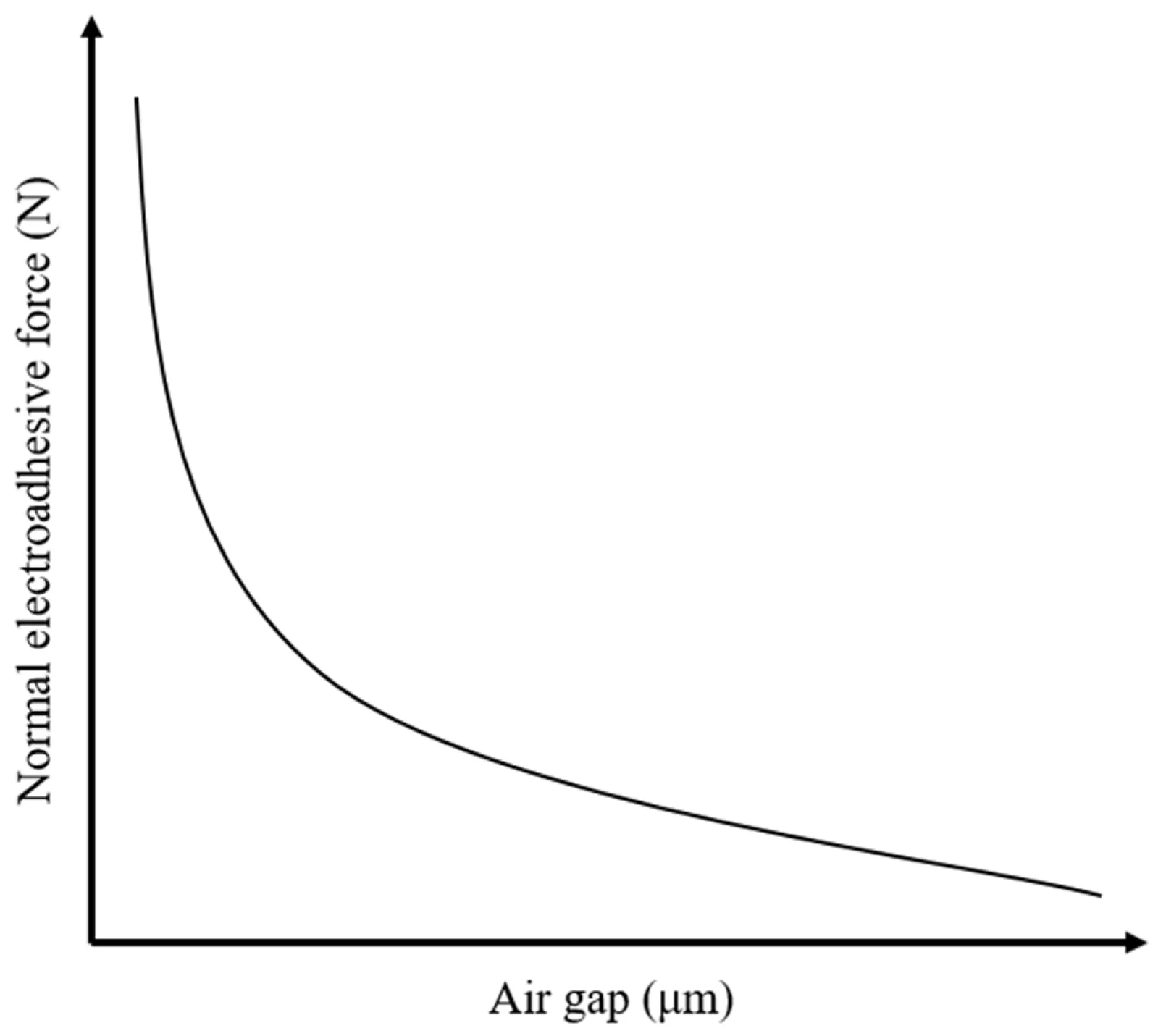
Typical curve of the influence of the air gap on the electroadhesive forces.

In the modeling section it was shown that electroadhesive models take into account the influence of the air gap between the electroadhesive and the substrate. It has been confirmed in [51,59] that the air gap has a significant effect on the electroadhesive forces arising between the electroadhesive and the substrate. The magnitude of these forces is inversely proportional to the air gap size. The results obtained in [51,59] are in good agreement with the theoretical calculations presented in [18,37]. A typical curve of the influence of the air gap on the electroadhesive forces is shown in Figure 20.

## 6. Technology Integration

The main drawback of electroadhesive systems is the relatively low electroadhesive force. Therefore, a logical solution is to attempt to combine electroadhesive technology with other adhesive technologies. Currently, the most common approach is the development of hybrid dry adhesives that combine the properties of two adhesion theories: mechanical adhesion, where the surface microrelief ensures tight initial contact between the adhesive and the substrate surface (gecko-like adhesion), and electrostatic adhesion, where the generated forces increase with closer contact. Thus, a synergistic effect is observed, resulting in increased adhesive bond strength.

**Figure 21:**
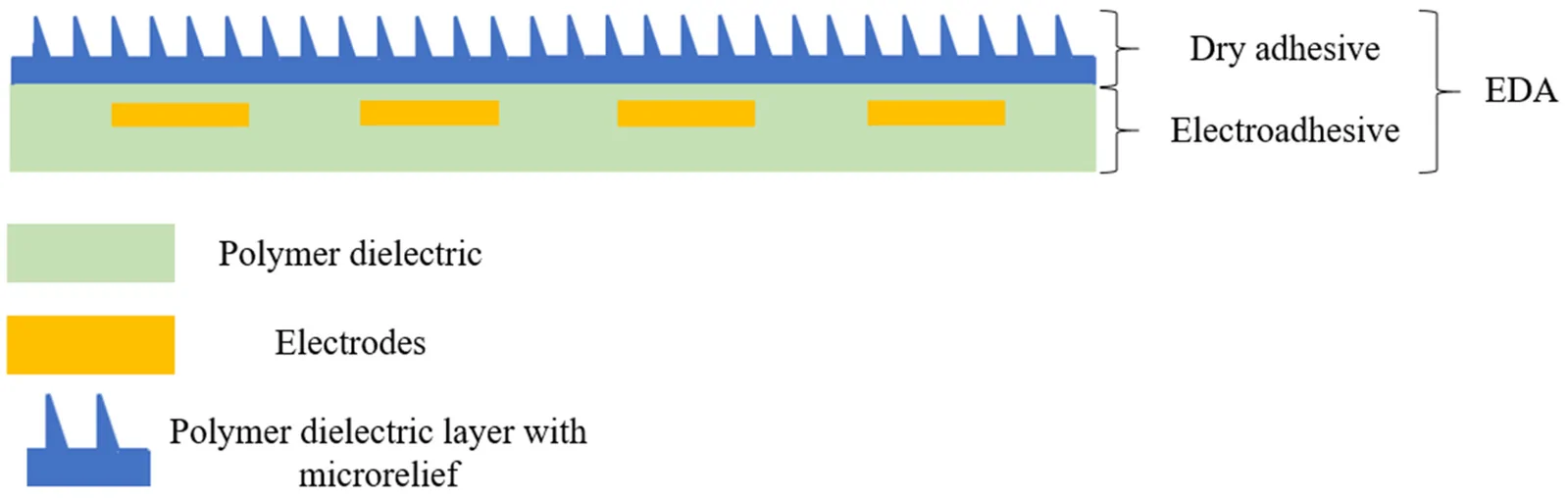
EDA design.

In [27,59], attempts were made to produce similar hybrid adhesives that combine surface microrelief with electrostatic attraction (electrostatic/dry adhesive—EDA). The design of such an adhesive (EDA) is virtually identical to that of an electroadhesive, with the only difference being the specified surface microrelief. A schematic of the EDA design is shown in Figure 21.

In [27], a group of researchers developed various types of adhesives, including dry microrelief adhesives, electroadhesives, and hybrid EDA systems. Their goal was to determine the maximum shear loads these adhesives can withstand. Tests were conducted on various materials, such as glass, wood, steel, graphite, plasterboard, polyimide, and various types of ceramics. Numerous experiments showed that on perfectly smooth surfaces, electroadhesives demonstrate the best results compared to dry and hybrid adhesives, achieving the highest shear stresses. This is due to the fact that a smooth surface ensures tight contact, a necessary condition for maximum electrostatic adhesion efficiency. However, on all other surfaces, including rough ones, hybrid EDAs exhibited the highest shear forces. This is achieved thanks to the flexible dielectric fibers, which form a microrelief that ensures the tightest possible contact between the adhesive and the substrate. As a result, the electrostatic component of the adhesive forces in such a bond reaches its maximum.

Thus, the creation of hybrid EDAs seems more than justified, and further research in this direction may be very promising.

## 7. Application and Future Perspectives

Currently, numerous devices have been developed using the electroadhesion effect. The greatest interest in this effect is observed in the following areas: robotics (all kinds of robotic grippers, climbing and crawling robots); microelectronics (powering electroadhesives using triboelectric nanogenerators (TENG), various touchscreen displays); the space industry (docking devices, special gloves and soles for astronauts); and biomedicine. This section discusses the application of electroadhesion in these industrial areas.

### 7.1. Robotic Grippers

Robotic grippers are an integral component of a variety of autonomous materials handling systems [63]. Among these, robotic grippers operating based on the electroadhesive effect, known as robotic electroadhesive grippers, stand out. These devices offer a number of unique advantages, particularly when handling fragile, flexible, and porous materials on assembly lines. The best-known robotic electroadhesive grippers to date are described in [64,65,66,67,68,69,70]. Also, the study [34] provides a classification of existing robotic electroadhesive grippers based on the degree of deformability of their working surface:

- Rigid electroadhesive grippers;
- Flexible electroadhesive grippers;
- Stretchable electroadhesive grippers.

Rigid electroadhesive grippers are designed for handling large, flat objects with a significant surface area. High structural rigidity ensures stable weight retention, but such devices are ineffective when working with objects of complex geometric shapes due to the inability to ensure tight contact. Flexible electroadhesive grippers are used to overcome these limitations. Their flexing ability allows them to adapt easily to curved surfaces, enabling interaction with a wide range of objects, including parts with convex or concave profiles. Stretchable electroadhesive grippers offer the greatest versatility. Their key feature is their ability to actively deform and change their geometry directly during operation. This allows the gripper to literally “wrap” objects of complex, variable shapes, significantly increasing the effective contact area and the strength of the electroadhesive interaction. This adaptability makes them indispensable for the precise manipulation of fragile or non-standard components in dynamic environments.

### 7.2. Climbing and Crawling Robots

Climbing and crawling robots are unique mobile devices that can adapt to a variety of surfaces and landscapes in both two- and three-dimensional space. These devices are capable of effectively performing assigned tasks and could become indispensable assistants in dangerous or hard-to-reach areas, such as nuclear power plants or pipelines, where there is a potential risk to human life [71,72]. The design and prototyping process for climbing and crawling robots involves three key steps [34]:

(1) Selecting a reliable adhesion mechanism that allows the robot to adhere to various surfaces;
(2) Selecting a flexible locomotion mechanism that allows it to move on various surfaces at various angles;
(3) Developing an energy-efficient actuation and control system to activate the locomotion functions.

Following Yamammoto’s publication in 2007 [73], electroadhesives began to be used in active support panels for crawling and climbing robots. A classification of such robots is presented in [34]:

- Tracked;
- Legged-1D (one-direction movement);
- Legged-2D (planar or two-direction movement).

Tracked robots operating on electroadhesion can achieve the highest speeds, but they have significant limitations in overcoming obstacles. To achieve turns, these robots use double tracks. Typical tracked robots using electroadhesion include the devices presented in [73,74,75,76,77].

“One-legged” electroadhesive robots (legged-1D) move by sliding two or more electroadhesive panels in a single plane. Another variant involves connecting a linear artificial muscle to two such panels, forming a pair of paws. However, such robots have speed limitations and cannot freely navigate cracks and other obstacles. Examples of such “one-legged” robots can be found in [73,74,78].

In “two-legged” robots (legged-2D) based on electroadhesion, the panels attached to the legs are separated from the drive components. These robots move like caterpillar worms, allowing them to traverse more complex terrain, such as chasms, cracks, and obstacles. However, their speed is limited. Typical “two-legged” robots based on electroadhesion are presented in [71,75,79,80,81,82,83].

In [84], a robot was developed that combines two vacuum-driven springs for sliding and turning, as well as two electroadhesive panels that act as legs. This robot demonstrated impressive results, developing a fairly high lifting speed (0.049 body lengths per second) and the ability to overcome narrow spaces and gaps (0.15 body lengths). Manipulating such robots based on the electroadhesive effect is quite easy due to the unique ability of this effect to adapt to various surfaces, which significantly simplifies the process of monitoring and control.

The work [79] presents a robot controlled by a dielectric elastomer actuator, capable of adapting to various environmental conditions, as well as different surfaces and inclination angles.

### 7.3. Haptic Technologies

Haptic technologies refer to methods that simulate the sensation of touching a surface by artificially stimulating mechanoreceptors in human skin. This is typically achieved through mechanical means, such as ultrasonic vibration [85], which physically stimulates the skin, or through electrical stimulation targeting subcutaneous nerve endings. Haptic devices, including touchscreens and displays, are being actively developed for portable consumer electronics like smartphones and tablets. They also find applications in human–robot and human–computer interaction systems [86].

There are two methods for using the electroadhesion effect in tactile feedback for touch displays:

- Electrovibration [87,88]—using alternating current voltage with different amplitudes, frequencies, and waveforms;
- Electroadhesion [89,90]—using direct current voltage.

Both methods of creating tactile sensations rely on friction between a person’s finger and a touchscreen. In both cases, a voltage is applied to the touchscreen’s conductive layer, causing an electrostatic attraction between the finger and the screen.

In the case of electrovibration, alternating current stimulates tactile receptors in the finger: Merkel discs, Ruffini corpuscles, Meissner corpuscles, and Pacinian corpuscles. By varying the parameters of the alternating current, it is possible to stimulate different receptors and create a variety of tactile sensations.

In the case of touchscreens, direct current affects the sensation of friction between the finger and the surface. This requires the user to move their finger to perceive the tactile effect. A theoretical model of contact between a human finger and a touchscreen based on the electroadhesion effect is presented in [90], and a capacitive touchscreen is developed.

The works [91,92] present tactile touchscreens based on electrovibration for perceiving surface texture and roughness. The authors of the works [47,93,94,95,96] developed several variants of contact electroadhesive surfaces for “artificial fingers” that allow tactile sensation on touchscreens and displays.

In addition to touch technologies, tactile displays are also important devices for use in virtual reality. Currently, there are many types of tactile displays based on various technologies: DC motors, shape memory alloys, piezoelectric elements, pneumatic and electrostatic (electroadhesive) actuators [97,98,99]. The advantage of tactile displays based on electroadhesives [97,100] is their relatively low cost and higher resolution (clarity).

### 7.4. Triboelectric Nanogenerators in Electroadhesives

A triboelectric nanogenerator (TENG) is a transducer that converts mechanical energy into electrical energy through contact electrification and electrostatic induction [101]. When two materials, typically dielectrics, with different surface potentials come into contact, their potentials reach equilibrium. At this point, several electrons are released, leading to the generation of electricity. When the surfaces are separated, each seeks to restore its original surface potential balance. This causes the transferred electrons to rush back to their original surface through an external circuit.

Due to the high resistance of these devices, TENGs are capable of generating significant voltages, reaching kilovolts, with currents of only a few microamps. This makes them particularly attractive for use in low-power electronics [102,103], including powering electroadhesives, eliminating the need for bulky batteries.

In [104], TENGs were used for electroadhesion, increasing the voltage by more than tenfold using a mechanism for replenishing stray charges. In this study, aluminum foil and polytetrafluoroethylene (PTFE), which exhibit a high surface potential difference, were used as positive and negative triboelectric materials for the TENGs. Several TENGs were combined into a single block with a high output voltage, and the electroadhesive was placed on the surface of this block.

In an experiment, researchers in [104] succeeded in increasing the output voltage from 241 to 4750 V by introducing a channel for increasing the triboelectric charge, which was a high-voltage diode. These values are sufficient to power electroadhesives. This research provides a significant boost in the field of fully autonomous electroadhesive systems.

### 7.5. Space Industry

Space is one of the most hostile environments, posing a serious challenge to modern science and technology. Extreme operating conditions are determined by a combination of microgravity, deep vacuum, sharp temperature fluctuations, and harsh ultraviolet and radiation exposure.

Designing and developing electroadhesive systems for operation in such conditions requires careful consideration of each of these factors. Despite the complexity of implementation, technologies developed using electroadhesive systems open up broad horizons for the aerospace industry, finding application both in the interior of spacecraft and in outer space. Currently, electroadhesive systems in space have potential for application in the following tasks:

- Docking operations—the authors of [105,106] used electroadhesive systems for a controlled method of docking spacecraft and satellites;
- Orbital debris capture—the work [107] presents an adaptive capture based on the electroadhesion effect, designed for capturing orbital debris by spacecraft;
- External and internal movement in the spacecraft—in the work [108], the authors used electroadhesive systems to move the robot in zero-gravity conditions;
- Manipulation of objects inside spacecraft—the authors of the work [109] used robotic grippers based on the electroadhesion effect to pick up and place various objects in the simulated environment of a spacecraft;
- Astronaut equipment—the production of special gloves and shoes with built-in electroadhesive systems that allow astronauts to move around the skin of spacecraft and also have the ability to grip various objects.

### 7.6. Biomedicine

Electroadhesion is considered a promising approach in biomedicine for creating soft, controllable, and reusable fixation and gripping systems that can operate without mechanical fasteners or adhesives. This method utilizes an electric field to generate attractive forces between the device surface and biological tissue or medical materials, enabling precise control of contact, pressure distribution, and release moment.

A group of researchers [110,111,112] demonstrated the potential of using electroadhesive technologies for fixing hydrogel coatings of various forms based on natural anionic/cationic polysaccharides (chitosan, alginate, hyaluronic acid, pectin, etc.) to various plant, animal, and human tissues. This approach opens up fundamentally new possibilities in surgical practice. In particular, it enables the implementation of a technology for sutureless closure of surgical incisions, ensures atraumatic sealing of internal organ injuries, minimizes the risk of postoperative complications associated with mechanical suture material, and reduces the time of surgical interventions by simplifying the fixation procedure.

Initially, in works [110,111], the studies were carried out exclusively on the tissues of cattle; however, in work [112], the experiments were carried out on the tissues of plants and various species of animals, including mammals, birds, fish, amphibians, insects and worms, as well as on human tissues.

Researchers in [112] were able to detect a significant difference between the contact adhesion and electroadhesion of hydrogels to arterial, muscle, and cartilage tissues. However, no significant difference was observed in the case of adipose and brain tissues. It was concluded that for a significant difference to be observed between the contact adhesion and electroadhesion of hydrogels to tissues, the tissues must contain anionic polymers (e.g., hyaluronic acid, mucins, and pectin) located at the interface between the hydrogel and tissue. It is also worth noting that voltages in the order of kV are typically used in the context of electroadhesion; however, in these studies, electroadhesion was observed at a constant voltage of only 10 V.

For ease of understanding, all the above-mentioned areas of application of electroadhesion are collected in a common Table 4.

## 8. Conclusions

The scientific community worldwide is currently showing deep interest in the phenomenon of electroadhesion, driven by its enormous potential for high technology. Extensive work has already been completed on fundamental modeling of this effect: the nature of electric field propagation depending on electrode geometry has been studied in detail, and optimal electrode configurations for generating maximum adhesion forces have been determined. Electromechanical models developed to date allow for highly accurate prediction of normal and shear electroadhesion forces for various classes of materials.

Nevertheless, the persistent gap between theoretical calculations and the behavior of real systems dictates the need to expand the experimental base. Particular attention should be focused on studying how the molecular and supramolecular structure of polymers determines the electroadhesive response. A thorough understanding of these mechanisms will allow us to refine existing models and move from empirical research to accurate engineering predictions of interaction forces.

Parallel to theoretical advances, the practical application of electroadhesive devices is rapidly expanding. This technology is becoming the foundation for breakthrough solutions in a wide range of fields: microsystems engineering, robotics, the creation of autonomous electroadhesive systems based on triboelectric nanogenerators, haptic technologies, the space industry, and biomedicine.

Continued interest in electroadhesion confirms its status as a promising area of polymer materials science. However, despite their advantages, electroadhesive systems have significant limitations: the need for high operating voltages (1–10 kV) increases the risk of dielectric breakdown, especially at elevated humidity; adhesive strength remains significantly lower than that of traditional adhesives, limiting their use in load-bearing structures. Addressing these issues requires the development of new polymer systems with improved dielectric properties. That is why, further comprehensive studies of polymer structures and their behavior under various conditions will not only allow for a deeper understanding of the nature of this complex physical and chemical process but also the creation of a new generation of intelligent and safe adhesive systems capable of revolutionizing technology and medicine.

## Figures and Tables

**Table 1:** Adhesion theories and their description.

Theory	Description	Sample	References
Mechanical	The adhesive bond is formed as a result of mechanical interlocking of particles due to the roughness of the surface of the bodies	“Velcro” Gecko feet	[2,3,4,5]
Adsorption	The formation of an adhesive bond is due to the adsorption of adhesive molecules by the substrate surface	Pressure-sensitive adhesives on bonded surfaces Sealants	[2,3,6,7]
Chemical	The adhesive bond is formed as a result of a chemical reaction between the adhesive and the substrate	Use of silane coupling agents for fixing polymers on glass or metal surfaces	[2,3,8,9]
Electrostatic	The adhesive bond is caused by electrostatic attraction forces	Dust on the surface of objects	[2,3,10]
Diffusion	Formation of an adhesive bond by diffusion or interdiffusion of adhesive and substrate molecules	Connection of compatible or partially compatible polymer materials in viscous and/or highly elastic states	[2,3,11]

**Table 2:** Differences between the Nakamura and Yamamoto model and the Johnsen–Rahbek model.

Comparison Criterion	Johnsen–Rahbek Model	Nakamura and Yamamoto Model
Accounting for mechanical behavior	Not taken into account	Taken into account through the spring-damper model
Dynamics at constant voltage	Explains only the increase in strength	Explains both the increase and decrease in strength
Application	Simple estimation of force under constant stress	Dynamic characteristics control, experiments with control

**Table 3:** Main types of electrode designs and their characteristics.

Type	Electrode Geometry	Advantages	Disadvantages
Concentric circle	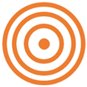 a system of nested circles with alternating potentials (the center is one pole, the rings are the other)	uniform force along the azimuth convenient for round/cylindrical objects easy to calculate capacity and force	reduced efficiency at the edges of the work area limited flexibility for irregular shapes
Square spiral	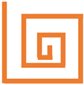 a single-wire coil with a square turn profile, often with a central terminal	compact placement on a rectangular site smooth increase in the field towards the center compatibility with planar technology	anisotropy of force (stronger along the axes of the spiral) difficulty in accurately modeling edge effects
Gilbert curve	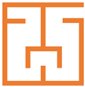 a self-similar polygonal line with multiple breaks that fills an area	large effective perimeter length with a small area enhanced adhesion due to field concentration at fractures resistance to local defects	labor-intensive manufacturing (photolithography/etching) increased leakage currents due to dense topology
Comb	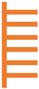 alternating “fingers” of two electrodes, nested one inside the other	maximum force per unit area fast response scalable (from µm to cm)	risk of breakdown at small gaps sensitivity to alignment accuracy
Interdigital	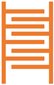 parallel pins of two electrodes oriented towards each other	adjustable frequency and step good repeatability of parameters compatibility with printing technologies	force anisotropy (mainly along the axis of the pins) weakening of the field in the center of the long pins

**Table 4:** Application of electroadhesion.

Field	Application	References
Robotic grippers	Microelectromechanical systems (MEMS) manufacturing	[64,65,66,67,68,69,70]
Robotics	Climbing and crawling robots	[71,72,73,74,75,76,77,78,79,80,81,82,83,84]
Haptic technologies	Displays and interfaces	[47,85,86,87,88,89,90,91,92,93,94,95,96,97,98,99,100]
Triboelectric nanogenerators	Autonomous electroadhesive systems	[101,102,103,104]
Space industry	Docking space stations, catching space debris, moving inside and outside a spacecraft	[105,106,107,108,109]
Biomedicine	Seamless connection of damaged tissues and internal organs	[110,111,112]

## Data Availability

Data are available upon request to the authors.
